# Development of a Hospital-at-Home Digital Twin for Patients With Frailty: Scoping Review

**DOI:** 10.2196/81510

**Published:** 2025-12-10

**Authors:** Faiza Yahya, Matthew Cooper, Wahib Saif, Mohamad Kassem, Hamde Nazar

**Affiliations:** 1 National Institute for Health and Care Research (NIHR) Newcastle Patient Safety Research Collaboration, Newcastle University, King George VI Building, King's Gate, Newcastle upon Tyne, NE1 7RU, United Kingdom, 44 1912086000; 2 School of Architecture and Built Environment, Northumbria University, Newcastle upon Tyne, United Kingdom; 3 School of Engineering, Newcastle University, Newcastle upon Tyne, United Kingdom

**Keywords:** hospital-at-home, frailty, digital twin, artificial intelligence, machine learning, risk prediction, digital health

## Abstract

**Background:**

Increasing demand on health care systems requires innovative, transformative solutions for efficient, high-quality care. One promising approach is digital twin (DT) technology, which leverages real-time data to create dynamic, virtual representations of a physical entity (individuals or space) to anticipate future scenarios and support care decisions. Although DTs have been explored in various sectors, their application in hospital at home (HaH), which delivers acute-level care in home environments, remains unexplored.

**Objective:**

This review bridges a critical knowledge gap, examining existing evidence on DT-enabling tools to manage patients with frailty in home settings. This will identify the underpinning architectural components required to inform an HaH-DT system supporting clinical decision-making.

**Methods:**

We searched 6 electronic databases and gray literature for primary English-language studies published between January 2019 and September 2025. Included studies reported on the monitoring or management of patients with frailty within their own home. Information was charted on a predefined data collection form to answer the research objectives. Review articles, protocols, and conference abstracts were excluded.

**Results:**

We included 69 reports: 54% (37/69) used quantitative approaches, and 36% (25/69) were pilot or feasibility studies. Reports were analyzed for DT-enabling tools and systematically mapped across the proposed 5-layered DT architecture: sensing, communication, storage, analytics, and visualization. DT layer taxonomies, interconnections, and classifications of data types collected (eg, about the patient, home environment, use of medical equipment) are presented. This evidence identifies DT-enabling tools for a variety of functions and a range of sensing technologies (eg, wearable-based passive sensing, active physiological sensors, ambient sensors detecting motion or environmental changes). The most prevalent communication modes were wireless and network-based (36/112, 32.1%), the majority (12/36, 33%) using Bluetooth. Better understanding of data management, particularly secure storage, is required within local health care systems. The emerging potential of predictive and prescriptive analytics for risk prediction, clinical decision-making, or activation of alert-triggered health interventions by clinicians was mapped. Analytics methods are currently largely descriptive. Advanced methods such as prescriptive analytics for recommendations of an optimal course of action and diagnostic analytics that highlight why a situation has occurred are lacking. DT-enabling tools demonstrate patient-centered benefits, including enhanced motivation, reassurance, and personalized care. Concerns include device accuracy, user acceptability, and implications for carers and organizational workflows.

**Conclusions:**

This review is among the first to systematically map DT-enabling tools to inform a potential HaH DT for patients with frailty, organized by a 5-layered conceptual model. Understanding these architectural layers provides the foundations for stakeholders to advance research and development in areas where there are knowledge gaps and consider how an HaH DT can effectively operate within current health care systems. Leveraging technology-enabled care in complex home-based settings provides great potential to deliver safer, personalized, timely care.

## Introduction

Health care systems are facing growing pressures due to an aging population, multiple long-term conditions, and rising levels of frailty [1]. To address these challenges, innovative solutions that enhance productivity are essential [2-4]. In the United Kingdom, a paradigm shift is being promoted [5,6], emphasizing “predict and prevent” care, transitioning from “analog to digital” and delivering care closer to home [2-4,7]. One potential solution is hospital-at-home (HaH) services, sometimes called virtual wards [8], which deliver hospital-level care in a patient’s place of residence. These services aim to avoid hospital admissions or facilitate early discharge, demonstrably improving patient satisfaction, improving outcomes, and reducing admissions to hospital and residential care [8-10]. One area of use is for patients with frailty, who are at increased risk of functional decline and mortality [11,12]. The dynamic nature of frailty [11,13] underscores the need for timely detection, personalized care, and home-based interventions to support recovery.

In the United Kingdom, national guidance advocates the integration of digital technology to optimize service delivery [8]. National priorities have promoted significant investment in a ”tilt towards technology“ [3], with the UK public sector spending £26 billion annually on digital technology [2,14]. The revolution of Healthcare 4.0 [15-17] highlights the transformative potential of advanced technologies in a digitally connected world, especially in home-based care [3-6]. These technologies can empower patient self-monitoring; enhance clinical decision-making; and optimize resources, costs, and safety [4].

Reviews exploring technology in HaH care categorize these as low-intensity (eg, telephone or teleconferencing), high-intensity (eg, apps or wearables) [18], manual, or automated remote monitoring [19]. Evidence on how to fully harness these technologies to optimize clinical decision-making, enhance risk prediction, and personalize care is limited.

An emerging approach in health care is digital twin (DT) technology, which creates a dynamic virtual representation of a patient or health care system leveraging real-time data. DT aims to closely mirror the real-world counterpart, analyzing its parameters using sophisticated methods like machine learning (ML) for dynamic simulation and predictive capabilities [20]. These methods can aid timely decision-making. With a patient DT, clinicians can visualize current states and forecast future scenarios, such as deterioration, early risk identification, timely intervention, optimization of care, and resource allocation. This supports proactive rather than reactive health care. To develop a DT, collaboration is required across disciplines, including engineering, medicine, computing, and data science, to understand the required data and enabling technologies within the 5 DT architectural layers: sensing (capturing information), communication, storage, analytics, and visualization [20-23].

The potential of DT in health care is increasingly recognized—leveraging artificial intelligence (AI), Internet of Things (IoT), big data, and predictive analytics—to anticipate health risks, support early disease detection, and enhance operational efficiency [24]. DTs can simulate disease progression, enabling proactive, personalized treatment and outcome prediction [5,25]. Examples include electrocardiogram (ECG)-based heart rhythm classifiers to detect heart problems [26], drug interaction modeling to predict medication responses [27], and dynamic glucose monitoring for medication adjustment in diabetes [20,28]. DTs have shown promise in personalized medicine, hospital management, and surgery [20,29,30].

However, challenges remain, with existing literature presenting various definitions of DT architectures [5,26,27,31]. Although this gives us valuable insight, the lack of clarity and consistency in frameworks for DT health care applications can make it difficult for implementation and evaluation [26]. This review aimed to address the knowledge gap linking DT architecture with HaH care for patients with frailty, considering the complexity and acuity of this patient cohort. To our knowledge, no literature currently addresses the application of DTs in this context.

This review aimed to identify existing evidence supporting the development of DTs for HaH care for patients with frailty, addressing the following research questions:

1. What DT-enabling tools have been applied in home-based care for patients with frailty, and how can they be systematically categorized?
2. How are data collected, recorded, and transmitted (sensing, communication, and storage)?
3. What outcomes are evaluated, and how are the results being used to inform care (analytics/decision-making, visualization)?
4. Are the tools evaluated for effectiveness and, if so, by what methods?
5. What are the challenges and opportunities with adopting DT-enabling tools for HaH care?

## Methods

### Review Principles and Protocol

This scoping review followed the principles in the Joanna Briggs Institute (JBI) methodology for scoping reviews [32] and is reported in line with the PRISMA-ScR (Preferred Reporting Items for Systematic Reviews and Meta-Analyses extension for Scoping Reviews) [33] (Checklist 1) and PRISMA-S checklist (Checklist 2). This review followed a published protocol [34] and the 5 key methodological stages of scoping reviews [35]: (1) identifying the research question; (2) identifying relevant studies; (3) selection of relevant studies; (4) charting the data; and (5) collating, summarizing, and reporting the results.

A scoping review was identified as the most appropriate systematic, rigorous approach to knowledge synthesis to capture the required information, as no previous systematic reviews nor evidence exists to answer the research question [36]. Additionally, many primary studies and evidence in practice are heterogenous in nature, making a scoping review a useful approach to capture the breadth of information and map key concepts required for this work.

### Screening and Eligibility

#### Identifying Relevant Studies

Screening of eligible studies was conducted in line with a predefined protocol [34], and the search strategy was supported by a medical research librarian. We searched 6 electronic databases (Embase, MEDLINE, Cochrane CENTRAL, CINAHL, Web of Science, and Scopus; Multimedia Appendix 1), and all identified studies were uploaded to a Rayyan database for screening and deduplication [34]. Gray literature and local evaluation reports relevant to HaH in the United Kingdom were retrieved from National Health Service England, Department of Health and Social Care, and HaH Society websites; the FutureNHS Platform; and related links. Experts in the field and authors were contacted to identify any additional sources. Forward citation searching from the included papers was also conducted.

Primary studies published in the English language were included. To obtain the most up-to-date and relevant information, only studies published from 2019 were included. Initial searches were run until September 11, 2024, then re-run and updated up to September 16, 2025 (Multimedia Appendix 1). We also recognized that the COVID-19 pandemic may have vastly impacted patient care models and digital technology use; therefore, we wished to capture the most advanced information. Included studies had to report on the monitoring or management of patients with frailty within their own home environment and had to include the reporting of outcomes related to effectiveness, usability, acceptability, and safety. Review articles, protocols, and conference abstracts were excluded as they did not include the primary data required to meet the review objectives.

#### Selection of Sources of Evidence

In line with JBI guidelines [32], a pilot stage was conducted in which 2 authors reviewed a sample of 25 title and abstracts independently and any conflicts were resolved through discussion, allowing screening to continue. The primary researcher (FY) was responsible for full-text screening, with support from a second independent reviewer (MC, HN) to validate the appropriateness of inclusion and exclusion. Discrepancies were resolved through discussion among all 3 reviewers. During the screening phase, reviewers agreed that articles were excluded if the population did not meet the inclusion criteria, did not explicitly mention frailty or meet the definition of frailty as per the British Geriatric Society definition stated in the protocol [34], included older adults only or prefrailty only, or set out to assess frailty as an objective or outcome measure. Studies were also excluded if the setting was not a patient’s usual place of residence (ie, a nursing home).

### Data Charting

A data extraction form was developed in a format that answered the objectives of the research question covering evidence for each of the 5 architectural layers, as well as country, population, concept, context, applications of the tool, evidence of effectiveness reported, and barriers or facilitators. This was independently piloted with different types of evidence sources (eg, from databases or gray literature) by 3 reviewers (FY, HN, and MC) then collaboratively discussed to highlight and clarify any discrepancies [36]. It was highlighted that data extraction should focus on the tools used rather than the study or model of care itself. It was also apparent that information obtained from the evaluation reports could often be less structured and more ambiguous, making data extraction more challenging. Data extraction and charting from the included sources of evidence were done initially by the primary researcher (FY) with independent confirmation from the other researchers (MC, WS, MK, HN) to validate the findings. Any uncertainties were clarified via discussion after reviewing the original evidence source. If the required information regarding the tool was not available in the included source, the cited protocol or paper was retrieved for further detail to answer the research objectives.

### Critical Appraisal

Critical appraisal was not required in line with JBI guidelines for scoping reviews.

### Ethical Considerations

Ethical approval was not required for a scoping literature review.

### Collating, Summarizing, and Reporting the Results

Data were collated then summarized to identify themes and key concepts within each category in the data collection form. This was mainly (1) to map the extent and nature of the included studies and their characteristics and (2) to answer the research objectives (ie, the sensing technologies; the communication technologies; information storage, analytics, and visualization methods; Multimedia Appendix 2). Finally, the data collection sought to extract any evidence of effectiveness reported in the studies as well as any reported challenges and opportunities of the tools used to support our analysis and interpretation.

## Results

### Architectural Layers

**Figure 1.**
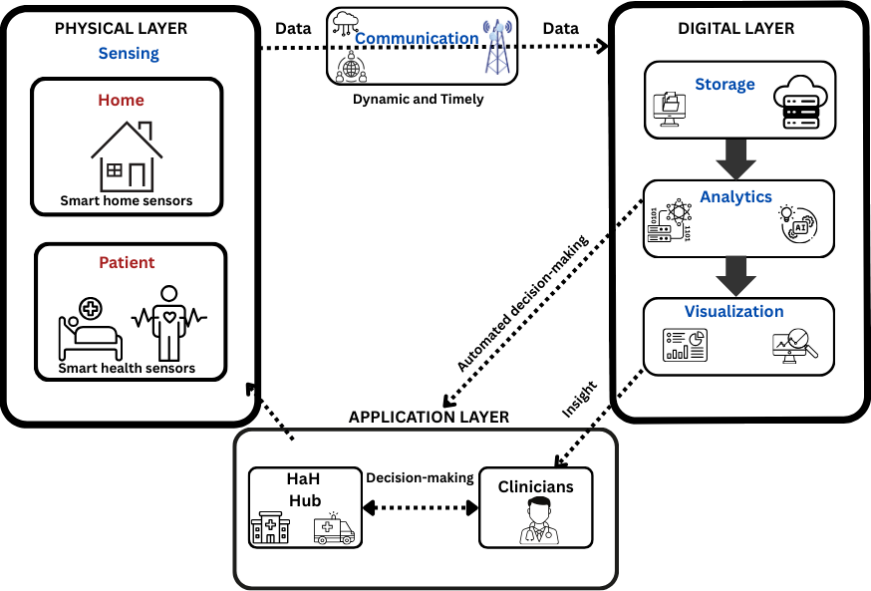
Conceptual model of proposed hospital-at-home (HaH) digital twin and architectural layers.

The results of this review found no existing DT in place for the management of patients with frailty at home nor HaH models. Therefore, in line with the objectives of this review, evidence on DT-enabling tools that could inform any of the 5 architectural layers (ie, sensing, communication, storage, analytics, and visualization) of an HaH DT was sought. These are visualized in the proposed conceptual model in Figure 1 and guided the categorization of the DT-enabling tools in this review based on their functionalities.

### Study Characteristics

**Figure 2.**
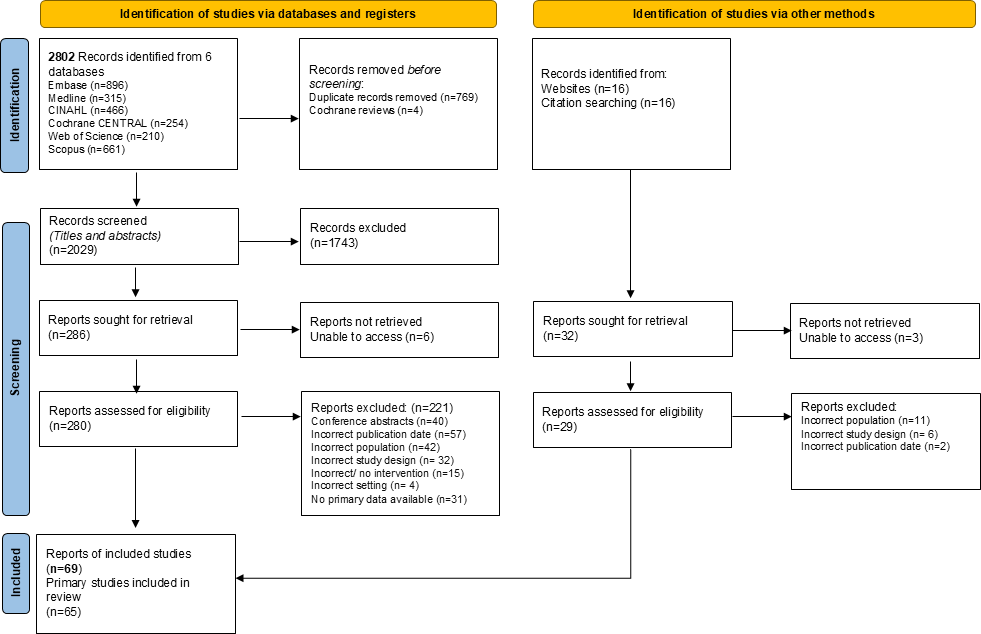
PRISMA (Preferred Reporting Items for Systematic Reviews and Meta-Analyses) flow diagram for study selection.

We included 62 reports from electronic databases and citation searches and 7 gray literature documents that focused on HaH care in the United Kingdom. As displayed in Figure 2, this resulted in a total of 69 reports (65 studies). Included studies spanned 19 countries across all continents: Europe (n=41), North America (n=17), Asia (n=9), Australia (n=1), and South America (n=1). Over one-half (37/69, 54%) of all included reports used quantitative approaches, followed by 27% (19/69) using mixed methods and 19% (13/69) using qualitative approaches. A large proportion (25/69, 36%) were pilot or feasibility studies testing the tools or intervention. Study designs were described as randomized controlled trials (n=16), cohort studies (n=5), longitudinal studies (n=5), observational studies (n=6), case studies (n=6), evaluations (n=7), feasibility studies (n=10), experimental studies (n=5), quasiexperimental studies (n=1), cross-sectional studies (n=3), or exploratory studies (n=5). Individual study characteristics can be viewed in Multimedia Appendix 3.

### Data Types and Applications of Existing Tools

**Figure 3.**
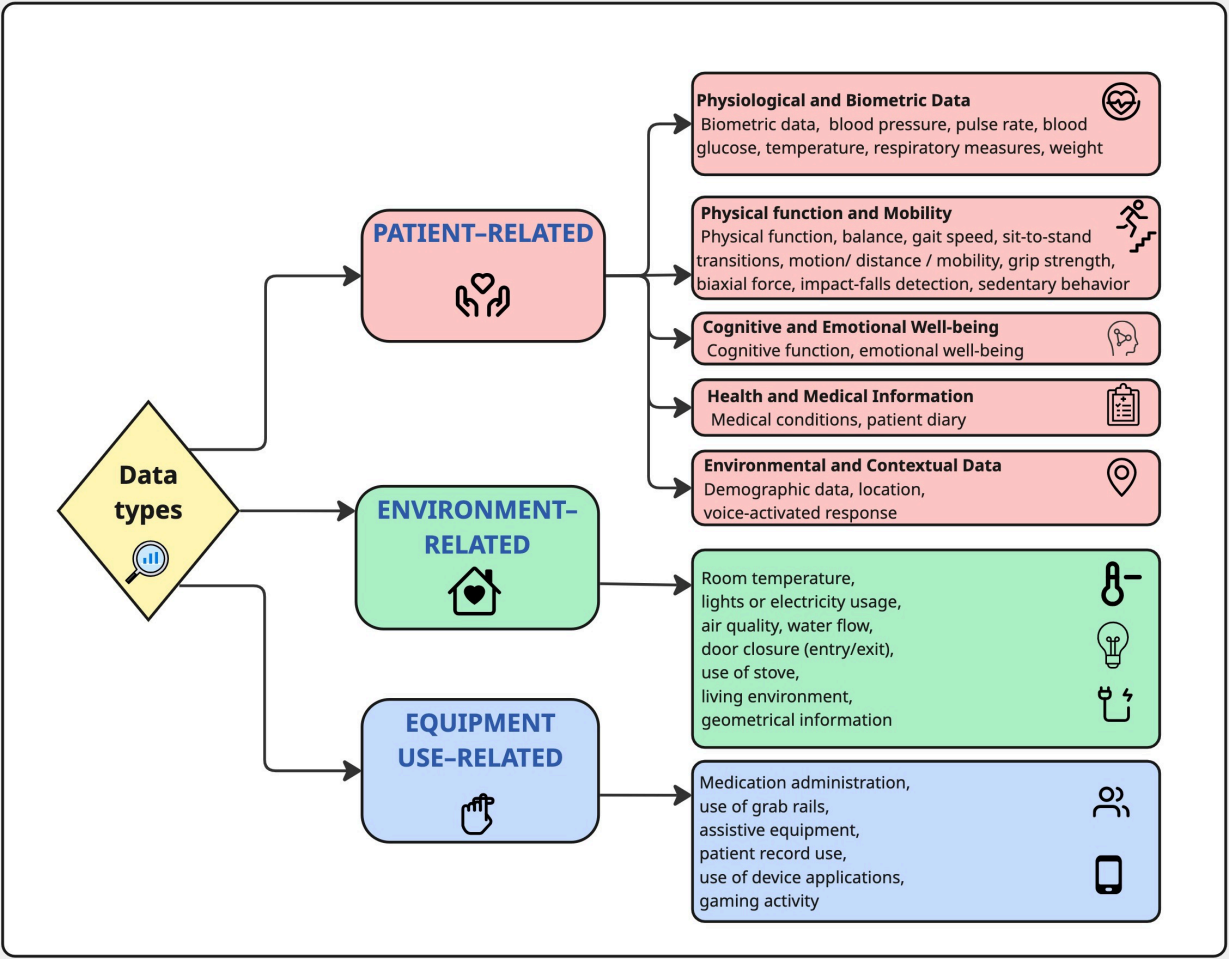
Types of data collected by the sensing devices.

The types of data captured by the tools (Figure 3) enable an understanding of health care management of patients at home, demonstrating that not only patient data but also environmental-related data and equipment use–related data can be collected. In addition to clinical monitoring, this can provide alerts about early changes in the patients’ usual patterns or behaviors such as the use of appliances in the home (ie, kettles), electricity usage, or use of medication (ie, in digital pill boxes). Many reports presented different tools with different applications and often a combination of functions. As displayed in Multimedia Appendix 4, a large proportion (36/69, 52%) of reports was focused on measuring frailty markers and the physical function of the patient. Similarly, tools were aimed at improving patient outcomes (n=27) or used for clinical monitoring and review (n=21). The alerting of early deterioration was also a function described in 14 reports; care communication and coordination (n=11) and monitoring of the patient environment (n=10) were also described. Only 6 reports described the use of tools for the education and training of patients (for example, exercise videos, self-care, or nutrition advice).

### Sensing Layer

The sensing layer is the fundamental layer within the DT architecture. It is crucial to understand how data are collected for the purpose of informing a DT. Data can be dynamic (changing over time) or static and collected using one or more data-collecting devices [21,23].

This scoping review included not only technological sensing devices but also any methods that were used to capture data that are used for patient care. Most of the studies relied on manual (human-assisted) methods, wearable sensors, or a combination of both to collect the required data. Manual data collection with equipment, via practitioner observations, or by patient self-reporting (ie, with a questionnaire or diary) was the most prevalent way of capturing data [37-66]. An array of wearable sensors existed to support with real-time data collection [55,57,63,67-70], vital sign monitoring [38,53,60,62-64,67-76], and assessing functional status [39,40,44,48,49,52-54,56,61,66-68,72,74,77-92] to assist with improving health outcomes [50,51,93-95] (see MultimediaAppendices 4  5).

Multimedia Appendix 4 displays the applications of the tools in relation to the included reports; however, Multimedia Appendix 5 provides a detailed mapping exercise of the applications used to the types of data needed to be collected and the technologies that could be considered within individual architectural layers.

**Figure 4.**
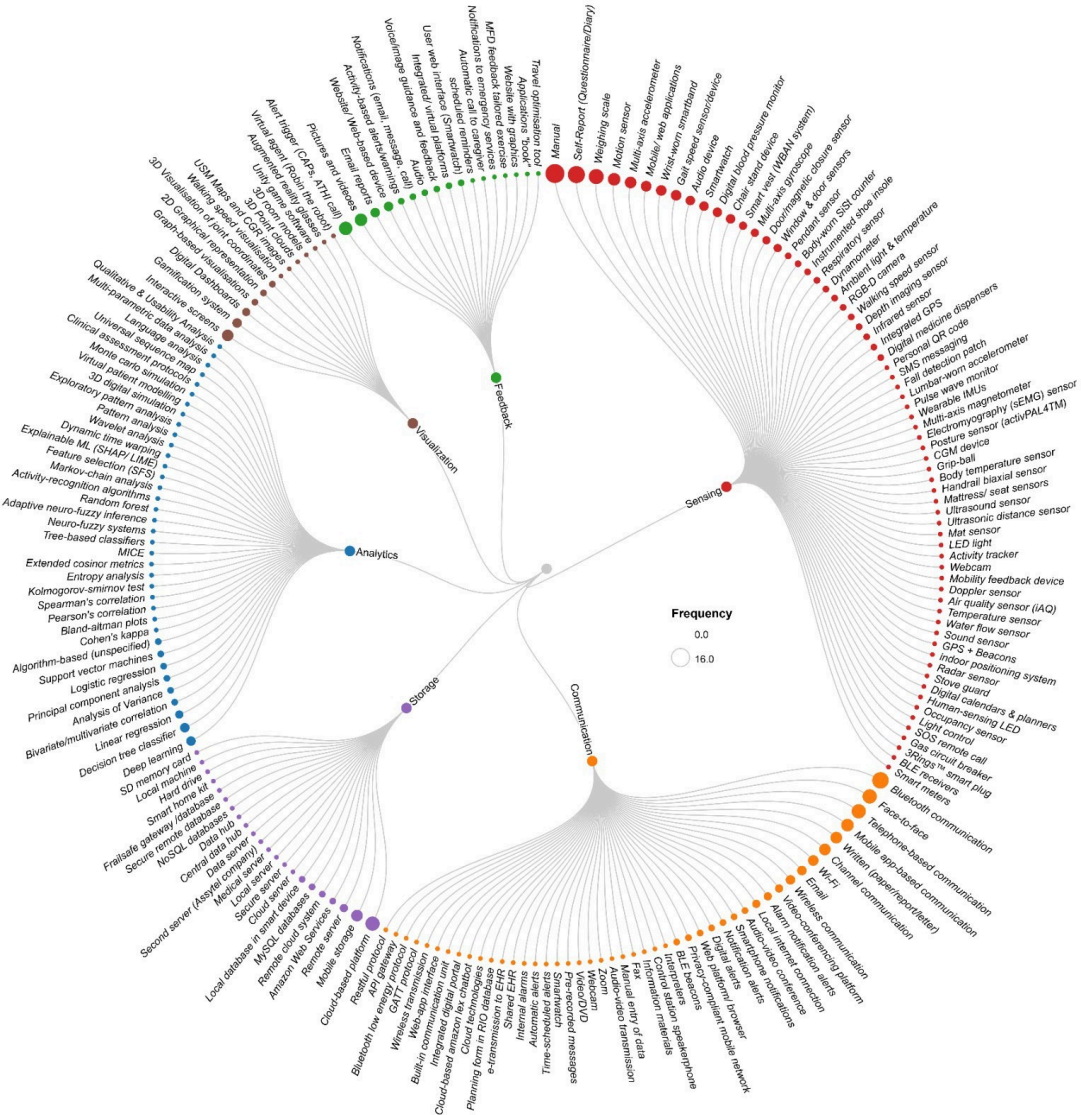
Distribution of all technologies in the included studies in accordance with the 5 layers of a digital twin: sensing technologies (green), communication technologies (orange), storage technologies (red), analytics technologies (blue), and visualization technologies (purple). The size of the circles represents the reporting frequency in the included studies. API: application programming interface; ATHI: alert-triggered health intervention; BLE: Bluetooth Low Energy; CAPs: clinical assessment protocols; CGM: continuous glucose monitoring; CGR: chaos game representations; EHR: electronic health record; GATT: Generic Attribute Profile; IMU: inertial measurement unit; LIME: local interpretable model-agnostic explanations; MICE: multiple equation chain interpolation or multiple imputation by chained equations; RGB-D: red green blue depth; SFS: sequential forward selection; SHAP: Shapley additive explanations; USM: universal sequence mapping; WBAN: wireless body area network.

Other types of sensors (listed in Figure 4), categorized as physiological measurement sensors (Table 1), are nonwearables or nonobtrusive and capture data by activation. Examples include smart weight scales [52,53,55,56,66,67,74], “grip balls” to measure grip strength [80], or devices that are used to measure gait speed [52,55,56,66]. Other nonwearables included ambient sensors that measured motion and activity. Examples included visual (ie, cameras, webcams) [73,86,87] or motion-sensing (ie, motion sensors, mat sensors for sedentary behavior) [54,67,80,86,87] devices. Some devices had inbuilt hardware that processed the raw data from accelerometers, such as Arduino Nano boards [83] used for local data processing, eliminating the need to transmit unprocessed data externally. Similarly, a custom smart speaker built on a Raspberry Pi platform included a 6-microphone audio board, allowing it to capture and process audio data locally [67].

Studies also reported on ambient sensors that monitored the environmental surroundings (eg, air quality, water flow, infrared, sound sensors, or GPS [54,68,86,87,96,97]). These would enable the monitoring of any change to usual patterns of behavior in the home. An additional category of sensing included assistive or safety technologies that would be related to patient welfare. This may include detecting any unusual entry or exit to the home by window or door sensors and detecting any hazards in the kitchen with stove guards or gas circuit breakers [68,86,87,96-98]. Detecting the use of electrical appliances in the home may involve sensors on kitchen appliances (ie, 3Rings smart plug) [98] to detect any unusual patterns alerting to deteriorating condition of the patient [87,98]. Digital calendars and digital medicine dispensers can provide reminders and detect medication administration [96]. Most studies used a combination of sensors to build a picture of the environment and the patient as part of their intervention.

**Table 1.** Distribution and categorization of tools and technologies in the included reports that showed positive outcomes.

Categories of tools and technologies	Results, n
Sensing	
Manual (human-assisted)	33
Wearables (passive) sensors	18
Physiological (active) sensors	21
Ambient (motion) sensors	8
Ambient (environment) sensors	1
Assistive and safety technologies	2
Communication	
Verbal communication methods	10
Written communication methods	10
Audiovisual technologies	2
Mobile and digital communication	13
Health Information Exchange and cloud-based	3
Wireless and network-based communication	26
Storage	
Cloud-based	5
Server-based	6
Databases	2
External storage	5
Analytics	
Statistical methods	9
Machine learning–based methods	10
Time series or signal processing approaches	3
Simulation and modeling approaches	2
Other	4
Visualization	
Graphical and dashboard	5
3D visualization	6
Software or hardware	3
Feedback	
Health alerts and notifications	17
Health feedback and reports	10
Mobile and digital platforms	9

### Communication Layer

The communication layer, sometimes referred to in the literature as the transmission layer or network layer, is responsible for receiving the raw data from the sensing layer and transferring it to the storage layer to be processed and visualized [21,23]. This layer acts as the intermediary connection or “the bridge” sharing information between the physical layer and the other components in the virtual layer [22,23,99]. In this review, communication methods are any methods used to communicate the information or transmit data, whether this is technology or nontechnology-based. This also included verbal, written, and audiovisual or multimedia as categories of data transmission.

However, digital tools have been shown to transfer data via 3 main categories: (1) mobile or digital messaging, (2) Health Information Exchange or cloud-based communication, or (3) wireless and network-based communication (see Figure 4, Table 1).

Bluetooth technology was used for the communication layer in 12 reports [52,55,62,66,70,72,74,78,83,89,92,100]. Bluetooth Low Energy (BLE) protocols were also used and are known to be more energy efficient than traditional Bluetooth [21]. Wireless networks such as WiFi were popular networks of communication in many cases, with some reports emphasizing that a local internet connection was not always required [87,101]. Nonetheless, studies also reported a privacy-compliant mobile network was needed [57,70]. Integrating a communication layer into the software or providing 2-way messaging channels were seen as useful for connecting clinicians and patients or caregivers [55-57,61,73]. A communication protocol is a key component of the communication layer that governs how data are transmitted between the system components, ensuring efficient and secure transfer across the communication network [21,22,27]. Different protocols were reported in the reviewed literature, including GATT (Generic Attribute Profile) protocols [72], RESTful protocols [82], or use of an application programming interface gateway [74]. Some sophisticated technologies used a cloud-based Amazon Lex Chatbot in smart speakers to manage conversations with users using automatic speech recognition in natural language [67].

### Storage Layer

The storage layer is responsible for hosting the sensed raw data from different sources as well as other integrated data such as historical data that can be used for forecasting future analysis [21]. Other studies included this as part of the network layer [23] or encapsulated it with the communication or analytics layer. We have presented this as its own layer for a more granular examination, to identify how data are stored, and to clarify what potentially needs to be considered to develop an HaH DT prototype.

As represented in Figure 4, the storage layer was the least described in the included reports (n=18). However, we categorized these as cloud-based storage solutions, server-based storage solutions, databases, or external storage solutions. The most used storage options described were cloud-based platforms [62,67,68,70,74,81,84,92,100,102], mobile storage (such as apps) [51,55,56,75,98,103], or remote servers [80]. Secure servers were described on either desktops or mobile devices [103]. Few studies described using secure Amazon Web Services as a storage method [52,74]. NoSQL databases [67] and MySQL databases [48,82] were also used, representing the complexity of the types of data in patient care at home.

### Analytics Layer

This layer is responsible for data preparation, processing, and translation to produce meaningful insights and can sometimes be referred to as the computing layer [21,23]. In the majority of the literature, this comprised all the data-driven models [23]. In the analytics layer, data may go through data modeling, data mining, or data fusion to consider the current physical state and manage big data to enable and represent future predictions [22]. Prior to analysis, the data may go through a stage of data aggregation. Data aggregation was mentioned in 7 reports [37,52,65,66,69,95,104], including measuring changes in clinical scales or multiparametric data analysis [74] to understand and collate the data retrieved from the sensors, although this would not constitute technical analysis itself.

In the analytics layer, methods could fall under any of 4 types: descriptive (*What has happened?*), diagnostic (*Why have we seen this result?*), predictive (*What might happen?*), or prescriptive (*What should we do?*). However, the included reports described methods corresponding to only 3 of the 4 analytics descriptions: descriptive, predictive, and less so prescriptive methods.

Descriptive analytics could encompass pattern analysis or a variety of statistical analyses as represented in Figure 4. For example, tests like Cohen kappa were used for categorical data from motion sensors, Bland Altman plots were used for continuous data (ie, sedentary time and stair climbing time), and bivariate correlation analyses were used for weight data [67]. In addition, principal component analysis has been used for dimensionality reduction with large datasets [74,102].

Predictive analysis allows forecasting of future possibilities and was described in 8 reports [39,49,54,58,67,74,85,87], primarily using ML algorithms. This could either be supervised ML (which uses labeled data to train algorithms) or unsupervised ML (which uses clustering on unlabeled data) [105]. Examples of supervised ML included methods of regression (ie, using logistic regression analysis) [67,85] or classification (ie, using tree-based classifiers [39,67,79] or support vector machines [67,74]). Reports also described the use of artificial neural networks, deep learning (multilayer perceptron neural network or with an artificial neural network architecture) [87], and neuro-fuzzy systems or adaptive neuro-fuzzy inference systems [87].

On the other hand, unsupervised ML would, for example, encompass neural networks or hierarchical cluster analysis like dynamic time warping distance matrix [81]. This can be a method of time-series analysis, which has been demonstrated to distinguish between ascension and descension on a stairway by measuring changes in speed and frequency and force on a handrail biaxial sensor [81].

One report [54] used universal sequence mapping as a method to model human frailty through smart home sensor readings. Alternative methods, namely approximate entropy and sample entropy, were compared for sensitivity using Monte Carlo simulation. Markov-Chain analysis was then used to understand which method had higher precision to understand behavioral complexity as a potential biomarker for frailty [54].

Prescriptive analysis evidence demonstrated this can support decision-making using methods such as modeling (ie, 3D digital simulation or virtual patient modeling [74]). One study also used social media analysis in their platforms to assess mental frailty or personality trait shifts (ie, with language analysis) to process the users’ typed text, social interactions, or questionnaires [74].

### Visualization Layer

This layer is a way of presenting the analyzed data and generating insights to enable informed decision-making by clinicians, patients, or caregivers. Some studies combined this with analytics as a “data analytics and visualization layer“ [22]; however, for the purposes of this study, we realized that these layers are primarily distinct. In this review, we also separated the visualization layer from the feedback mechanisms in a DT.

This layer was categorized in 3 ways: (1) graphical and dashboard visualizations, (2) 3D visualization, and (3) software or hardware.

The graphical and dashboard visualizations are represented in 2D form. Common methods involved digital dashboards either on a computer or mobile device [70,86] or remote telemedicine center [63]. One study also described the use of universal sequence mapping both in analytics and as a way of visualizing patterns similar to chaos game representations [54].

A 3D visualization, such as with point clouds, was used to represent 3D skeletal reconstruction by combining IMU data, RGB images, and depth measurements processed by neural networks to understand functional activity [39]. Similarly, visualization of joint coordinates using visualization tools such as Master Active Gestures ID can help assess sitting or standing behavior [87]. Alternatively, visualization of a virtual living room through an interface was possible [62].

Software or hardware, such as gaming systems, was used to visualize progress or weekly changes [106], for example with exercise or walking speed, and can incentivize physical activity with digital rewards or ranking points. Such systems may include augmented reality glasses [74] or interactive platforms. Interfaces for this software can be accessed through the web or mobile apps. Some integrated platforms such as in-built interfaces on a wall or television in the patient’s home can also include a virtual embodied agent (Robin the Robot) to support and tailor advice to users [62].

### Feedback Mechanisms

In addition to the visualizations described in the previous section, methods for feedback to the users (clinicians, patients, caregivers) were used and categorized into 3 groups: (1) health alerts and notifications, (2) health feedback and reports, and (3) mobile or digital platforms.

Health alerts and notifications include automatic alert triggers when a risk is identified. One example is alert-triggered health interventions, which raise an automatic call to the clinician’s hub if a high risk is detected according to its risk categorization [58]. Calls may also be actioned directly to the caregiver [73,86]. Alternative forms are notifications via email or text message or to the emergency services [73].

Health feedback and reports involve email reports or letters with health information or location [38,69,70,95]. Visual reports with videos or photos are also common [83,87,95]. Some reports have also shown voice or audio feedback [83] or used specific devices like mobility feedback devices for tailored information [48].

Mobile or digital platforms are also commonly used for feedback. Some are integrated with other platforms such as electronic health systems and some are via web interfaces or applications [78,83] (ie, via a smartwatch [72]).

As described in Table 2, 26 of 69 reports explored the user experience including acceptability and usability, especially as these tools were in infancy of development and implementation. However, only 4 reports explored the impact on care delivery, the system, or clinical outcomes. Where reports demonstrated positive findings, there was a predominant use of manual sensing measures and using mobile apps and self-reporting (Table 1). Nonetheless, there is an emergence of wearable technologies, including wearable accelerometers that can be wrist-worn, lumbar-worn, thigh-worn, worn in a smartvest, or worn as a pendant sensor. Devices for smart weight and blood pressure monitoring as well as devices that measure gait speed, sit-to-stand transitions, and movement in the environment were also prominent.

**Table 2.** Outcome measures for the tools as assessed by the included reports.

Categories or groups	Definition includes	Report references
Validity of sensing	Accuracy, reliability, (sensor specificity, sensitivity)	[39,58,67,73,74,79-81,85,87,102]
User experience	Usability, acceptability, satisfaction	[44,45,48,51,52,55,58-64,66,68,72,75,82,83,86,96-98,103,107-109]
Practicality	Feasibility and implementation, compliance or adherence, safety	[39,48,50,53,58,84,94,106]
Impact on care delivery	For example, nurses’ time, workflow	[58,64,69,107]
Impact on physical activity or well-being	Patient or carers, including functional decline and risk of emergency department visits	[46,48-50,56,61,90-92,95,100,108]

### Challenges and Opportunities

Studies reported many challenges with the use of the tools that would need to be considered in future work, but they also described many opportunities. Challenges in these studies were categorized into 4 themes: (1) patient or carer factors, (2) device factors, (3) external factors, and (4) organizational or administrative factors.

#### Patient or Carer Factors

Most studies identified various patient-related factors impacting successful adoption of digital tools. These included varying levels of digital literacy [38,59,69,97,100], previous experience with technology [52], technophobia [72], and a general preference for face-to-face interactions [50], all affecting acceptability. Perceived benefit [45,97], and willingness [50,71,80,84,85,110] to engage are critical yet were often influenced by poor understanding of the technology’s role, unclear instructions, or complex processes. Where self-reporting was needed, it was recognized that this can lead to underestimating or overestimating measures [40,94]. Physical or visual impairments can affect usability, including device readability or difficulties with touchscreens, multiple-tapping, and managing pop-up notifications [66,68,69,78]. Other medical conditions (eg, psychiatric or cognitive disorders, dementia) may affect memory or concentration, hindering device use [71,100,110]. Furthermore, articulation issues were found to hinder voice recognition by assistive robots and led to inadequate conversation [44].

Some patients found wearables uncomfortable and inconvenient [68,78,89], disrupting daily activities (eg, washing), or experienced disturbances from LED lights or main leads [88], potentially attenuating psychological and physical burden (ie, stress, sleep disturbance, anxiety, depression) [61,97]. Variabilities in-home routines can also affect the ability to individualize care [39]. Carers reported burden where patients required support and had difficulties navigating technology independently. They sometimes felt they had to be “on-call,” leading to increased expectations on their care, which reduced their autonomy [86]. Furthermore, notifications to caregivers can also cause frustration, affecting well-being [61,86].

#### Device Factors

Beyond device complexity for certain patient groups (eg, frail older adults), this evidence highlighted uncertainties with device accuracy [39,95] and potential for false readings [58,83,98]. Some studies reported difficulties with devices distinguishing between subtasks or detecting visitors, leading to noisy or biased spatial measurements [39]. There were also concerns that the accuracy may not have matched hospital devices (eg, ECGs) [95] or varied between standardized clinic or home settings (eg, the 6-minute walk test) [85]. Current devices also lacked validation for specific populations (eg, those with frailty), leading to potential inaccuracies in algorithms like step counts [85]. Short or inconsistent data sequences, storage limitations [54], and missing values [89] further compromised the quality of results. Concerns also included corrupt data, device failure, and malfunctions [53,84]. Frequent recharging was also reported as burdensome [67,68], and incompatibility of devices to synchronize with platforms used by health care professionals can limit practical use [62,77].

#### External Factors

External factors outside of the home, such as internet signal or power outages, can also affect notifications and data quality [39,77,86]. Factors in the surroundings such as the size, shape, and geometry of a patient’s home environment or furniture can affect the practical application of sensors or standard clinical tests such as walking speed between two points [39,80]. This can result in artifacts in the data, adding a layer of complexity, especially if visitors are present, making data evaluation difficult. The environment cannot always be anticipated (eg, availability of sockets or flat surfaces), making locations for the devices challenging [88]. There is also a higher degree of variability in the home, dependent on the patients’ motivation or daily occupational demands, which cannot be predicted. Some patients may also find sensors on their furniture visually unpleasant, such as iAQ sensors on the dining table [68].

#### Organizational Factors

The use of devices often requires efficient and secure communication and coordination between services. Challenges were observed with variable adoption and understanding when remote monitoring was suitable [75]. A degree of model reorganization was needed to ensure prompt responses as well as access to clinicians out of hours [42,57]. Factors such as staff capacity, planning, and managing administration (if paper was used) were some of the challenges encountered [71]. Interoperability with clinical systems, data quality, and coding were also problematic [62,70,77].

#### Opportunities

Studies reported that the tools offered positive motivation for users [59,67,82], improving physical activity [52,62,78,103,106] and self-management [51,61,69,97,103]. Furthermore, they provided a sense of reassurance and safety for patients and carers [76,86,97,98]. Opportunities for autonomous and continuous monitoring [39,54,88] as well as integrating data from multiple sensors can increase the accuracy of information [79,87] and personalization [50,59,60,72,90,95,106] and provide the potential to evaluate trends to enhance decision-support systems [58]. At a service level, these can support early identification and referral [37,68], support cost-effectiveness of services by reducing the number of clinical visits [53,96], decrease waiting times [94], and support scalability of services [55]. Furthermore, information sharing between services, clinicians, and for research [67] means that data can be used as a proxy for frailty markers [74,85], to create frailty assessment tools (ie, web applications) [77], and to support integrated and collaborative care (ie, supporting staff handovers) [43,45,69,71,110]. Ways to overcome practical challenges with devices were considered in some reports; these included avoiding recharging by using induction charging [78,88] or hiding devices in socially acceptable objects [80].

## Discussion

### Principal Findings

This review aimed to explore the existing evidence for the potential DT architectural components that are tools or functionalities currently used in the management of patients at home with frailty and presents a novel conceptual model of an HaH DT (Figure 1).

The model is supported by previous definitions of DTs expressing that data acquisition and feedback should be dynamic, be timely [21], and have bidirectional communication [21,27]. Prior studies describing DTs have typically focused on specific aspects such as body organs, drug manufacturing, or device and facility management rather than holistic management [111]. Emerging evidence shows great promise of DTs in home care; however, a lack of in-depth studies exist, making implementation difficult [31].

**Figure 5.**
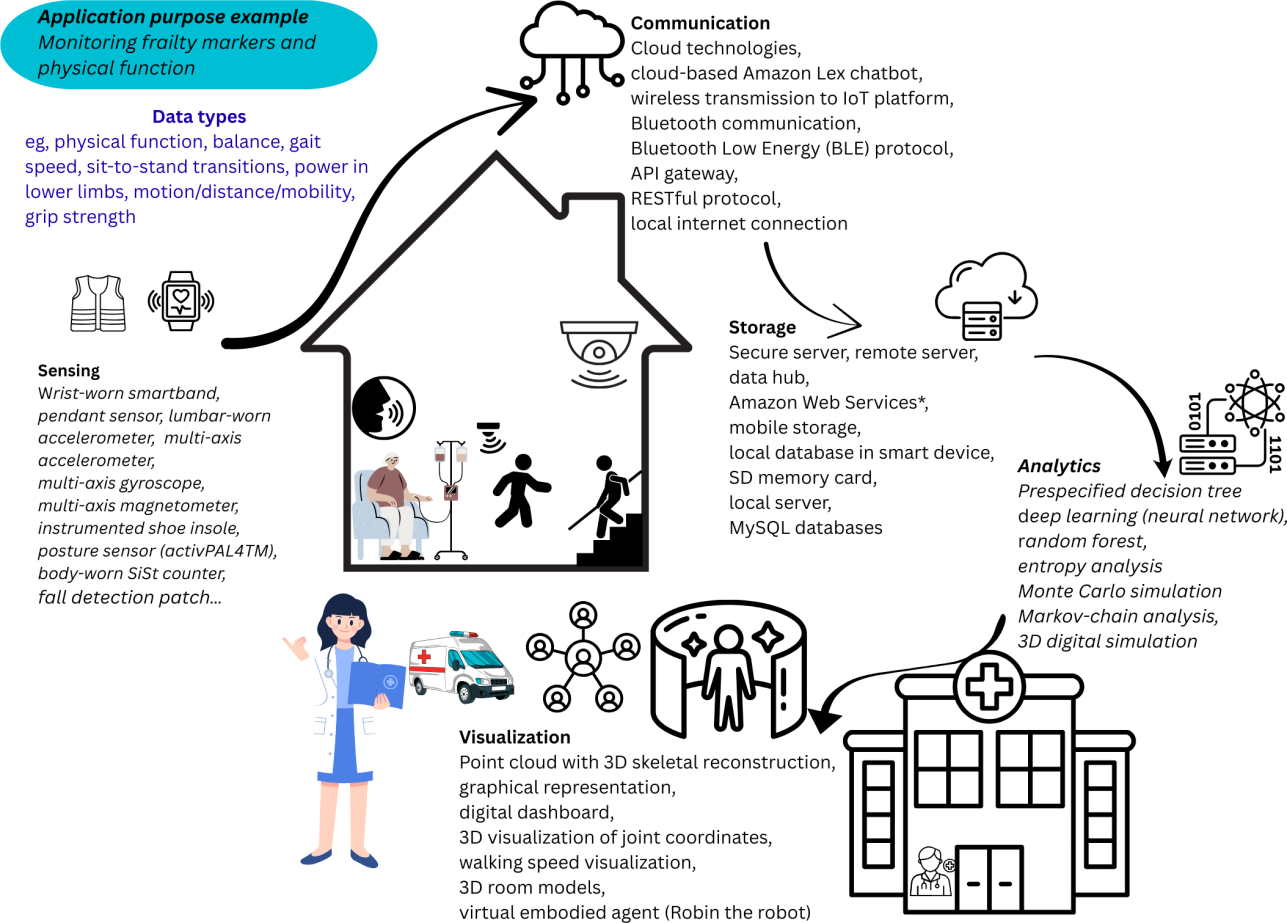
Example of possible use applications mapped to key digital twin (DT) architectural layers (see Multimedia Appendix 5 for full information). API: application programming interface; IoT: Internet of Things. *Although Amazon Web Services were mentioned in the included studies [52,74], it is important to recognize that other cloud computing services, such as Google Cloud or Microsoft Azure, exist and can be used [22].

The developed blueprint (conceptual model) presented in this review provides detailed information about the tools that can be used in an HaH DT and can be used to inform the development of a prototype for user testing. An example of a use application such as the monitoring of functional activity as a marker of frailty derived from the blueprint of an HaH DT detailed in Multimedia Appendix 5 is shown in Figure 5.

The results improve understanding so researchers, practitioners, and solution providers can better navigate the complexity of an HaH DT. This will help identify which data needs to be collected and how the data are managed in each layer, in relation to the specific applications.

This review recognizes that management as part of an HaH DT can support either at the service level (to support workflow management) or the patient level (ie, remote monitoring). Additionally, development of a DT in this context can be understood as a system of systems (ie, *comprised of* a DT of the home environment and a DT of the patient).

A taxonomy of existing sensing technologies has been identified that can be used both to monitor the patient and the environment, building a comprehensive picture of patient status and support decision-making. These offer the opportunity for collecting active and passive sensor data [112] (including the use of unobtrusive sensing technologies [113,114]), which can benefit the implementation of DTs for patients with frailty. This builds on current evidence around remote monitoring in HaH that aims for early prediction of patient outcomes [19,115]. In this review, communication technologies currently focus on Bluetooth and network-based wireless methods; however, the emerging use of advanced IoT technologies using 5G and AI may warrant further exploration.

### Data Governance, Security, and Ethical Considerations

Knowledge on efficient and secure data management (storage, integration, interoperability) with current health care systems remains crucial. Previous work has identified the need for data repositories such as ”data lakes” or data warehouses to enable data aggregation from multiple technologies and allow for data to be structured and analyzed in DTs [22]. Although the included studies demonstrated examples of either edge computing or cloud computing, health care systems are increasingly encouraging a cloud-first approach to improve efficiency and organizational agility and to enable scalability [116]. These may include public cloud services such as Google Cloud, Amazon Web Services, or Microsoft Azure [22]. Understanding the different data types from this scoping review and recognizing that data can fall into one of 3 types (structured, semistructured, and unstructured [22]) demonstrates the complexity of health care at home and the need for advancing knowledge around enhanced data management structures. Proposed DT frameworks suggesting data-centric approaches recognize that multimodal data integrating information from various health records are likely and potential solutions to aid implementation may involve in-silico studies using synthetic data [117]. Furthermore, the challenges with data acquisition, accuracy, and quality have similarly been highlighted previously [5,20,27,118]. In-depth information on data security was lacking in the included reports; however, some of the literature discussed potential solutions such as blockchain technology [5]. Data security measures need to be considered from development and inception, abide by security protocols and regulations, and consider aspects like robust encryption, authorization, and multifactor authentication to mitigate any risks of data leakage [119].

Other considerations such as fairness, accountability, transparency, and explainability when designing and implementing data-driven solutions and AI algorithms in a DT system should be integrated [120] . Cybersecurity when using IoT devices in health care remains a concern that can have serious consequences for the patient and the health care organization [121]. However, the integration of security into digital health is increasingly emphasized as we use more digital technology solutions. Carboni et al [121] discussed the balance needed between security and care practices, highlighting the perspective that health care systems in reality can be complex environments and solutions must involve end users rather than only the technology itself. Open conversations with users (both staff and patients) and participatory design approaches are methods to improve the adaptability and adoptability of advanced digital technology moving forward. Despite the potential of passive remote monitoring and DTs, previous work has likened remote monitoring to having “nanny cams” [101], highlighting that evidence and data are still required to promote their ethical use [5,31,101,120].

### Potential for Advanced Data Analytics

Most studies in this review described tools that used descriptive and predictive analytics, and some used prescriptive (clinical decision support). However, there is a need to explore the use of diagnostic analytics (ie, why is something happening?) by identifying anomalies in trends or performing root-cause analysis. In HaH, previous studies attempted to explore tools for risk prediction using predictive modeling, but these appear to primarily be case management tools and not necessarily dynamic nor real-time [122,123]. Emerging studies in health care have developed advanced risk prediction models to support decision-making in high-pressure, time-critical health care settings such as ambulance clinical transport decisions [124] . Understanding such novel approaches and how they integrate with electronic patient records is highly valuable [124]. Other examples [125,126] that also use ML algorithms in health care are emerging to alert to a patient’s deterioration early; however, these are hospital-based and may not reflect nonhospital settings. Health care is evolving, with increasing use of “command centers” to support patient flow and decision-making [127,128]. Although these update a dashboard in real time, they are often centralized in hospitals and still require manual input of information by staff. They can support workflow management and efficiency, aiming to enhance safety [127]; however, they may not have simulation or prediction capabilities like DTs. Moreover, their ability to adapt to complex home environments remains underexplored.

This review advances findings from other literature recognizing the potential of DTs in the management of hospital processes [20,127] to enhance efficiency or optimize environments [128]. For patient management, previous studies [20] have explored the potential of wearables as trigger warning alerts [20,27] that can be used in DT technology. In current practice in HaH, evidence for continuous vital sign monitoring using wearables shows benefit, and use is increasing in clinical practice; however, robust evidence is limited [19,115]. Adding to emerging evidence on DTs that are in early stages of development [118], this review demonstrates how existing technologies can be utilized in DTs specifically for HaH, as well as identifying key architecture that warrants further understanding, such as information storage, analytics, and visualization.

### Barriers to Adoption and Strategies for Integration

This review has provided some valuable learning regarding the barriers to adoption of digital technologies in the home for patients with frailty. These predominantly include digital literacy and understanding, both for patients and the workforce, followed by usability of the technology. Concerns around device accuracy, longevity, and interoperability and integration with current health care systems were also major challenges, which is recognized in some emerging studies related to remote monitoring [115,118]. Some potential strategies to overcome these would be to explore organizational readiness and ensure any prototype is co-designed with potential stakeholders (patients, carers, and health care organizations). Furthermore, improving awareness and understanding of the DT system to be deployed would be needed by factoring in training [129], funding, and workforce capacity.

### Strengths and Limitations

This review provides a systematic approach to reviewing current evidence on the tools used for the management of patients at home with frailty. As limited literature on HaH exists, this review was comprehensive enough to gather learning from the general management of patients at home. However, as the population in question was those with frailty, we may have excluded some studies that involved interventions that would have been useful to explore for nonfrail patients or those only at risk. During the search, many studies were correlation studies looking at the association of a parameter with frailty, and these were excluded as they did not meet the inclusion criteria. The search was comprehensive at trying to include all relevant search terms; however, we recognize that other search terms not included may have been helpful (ie, “Healthcare 4.0” or “5G”). Furthermore, the restricted inclusion of only those documents published in the past 5 years could have meant that helpful studies to answer our question may have been excluded.

### Implications for Future Work

This review has highlighted many of the technological capabilities for DTs in HaH as a first step to understanding the potential of current technologies in use for managing patients at home with frailty. This review provides the foundations to enable stakeholders to advance research and development in areas where there are knowledge gaps and consider how an HaH DT can effectively operate within current health care systems to enable safer, personalized, and timely care. However, to advance this concept further, we need to understand its potential application in current practice and its acceptability before it is developed and piloted. Using frameworks such as the Non-adoption, Abandonment, Scale-up, Spread, Sustainability (NASSS) framework [130] or the 6 categories in the DT consortium capabilities periodic table [131] would be helpful as a basis for understanding how the generated conceptual model may be implemented in practice. Furthermore, design of a potential HaH DT prototype and demonstration as a “show home” would benefit from stakeholder involvement and co-design to ensure that any advancing technologies are user-friendly, have supportive infrastructures, and have the appropriate educational support in place [129]. For organizations that wish to take learning from this review forward, understanding these 5 core architectural layers provides the fundamental knowledge to build on this foundation. Knowledge about how these interconnect within the current health care systems needs to be understood, especially with regards to data management and transfer of data between the 5 layers. Before this can be deployed in real health care settings, further research or small-scale prototype testing will be required, with a focus on a specific clinical cohort of patients and to determine which data types are appropriate for collection that would usefully alert to early deterioration and methods for coordinating these alerting systems or feedback. Evidence shows that a wealth of sensing technologies already exist, but understanding how these can be adapted to fit in with current systems is necessary. Inclusive methods for communication that do not rely on a patient to have connectivity are important to avoid digital exclusion. It would be useful to further understand secure data storage in health care or linking in with secure data environments before prototype development and the accuracy of potential predictive analytics.

### Conclusion

DTs in HaH offer a novel solution to prediction and prevention, reducing the burden on patients and health care systems and potentially improving clinical outcomes. Leveraging the use of technology-enabled care can enhance remote monitoring in real time to support clinicians with delivering care efficiently and safely. This review enabled us to understand the 5 architectural layers that can support stakeholders in advancing research and development of forward-thinking systems such as DTs, particularly within complex settings like HaH for patients with frailty. It also identified knowledge gaps in the existing evidence and allowed us to consider where health care systems should explore improvements to current structures to achieve successful adoption in a rapidly evolving landscape of digital solutions.

## Supplementary material

10.2196/81510 Multimedia Appendix 1 Search strategy.

10.2196/81510 Multimedia Appendix 2 Summary of data collection from studies according to digital twin (DT) layers.

10.2196/81510 Multimedia Appendix 3 Study characteristics.

10.2196/81510 Multimedia Appendix 4 Application(s) of the tool(s).

10.2196/81510 Multimedia Appendix 5 Mapping applications to digital twin (DT) layers.

10.2196/81510 Checklist 1 PRISMA-ScR (Preferred Reporting Items for Systematic Reviews and Meta-Analyses extension for Scoping Reviews) checklist.

10.2196/81510 Checklist 2 PRISMA-S (Preferred Reporting Items for Systematic Reviews and Meta-Analyses extension for Reporting Literature Searches in Systematic Reviews) checklist.

## Abbreviations

AI: artificial intelligence
DT: digital twin
ECG: electrocardiogram
GATT: Generic Attribute Profile
HaH: hospital at home
IoT: Internet of Things
JBI: Joanna Briggs Institute
ML: machine learning
NASSS: Non-adoption, Abandonment, Scale-up, Spread, Sustainability
PRISMA-S: Preferred Reporting Items for Systematic Reviews and Meta-Analyses extension for Reporting Literature Searches in Systematic Reviews
PRISMA-ScR: Preferred Reporting Items for Systematic Reviews and Meta-Analyses extension for Scoping Reviews
