# Supplementary material for: Development of a Hospital-at-Home Digital Twin for Patients With Frailty: Scoping Review
Source: J Med Internet Res. 2025 Dec 10;27:e81510. doi: 10.2196/81510 (PMC12694950; doi:10.2196/81510)
Supplement: Multimedia Appendix 1 [file jmir-v27-e81510-s001.docx]

**Searches for Scoping review:**

**Hospital at home digital twin for the management of patients with frailty**

Initial searches - Run 11.9.24

**Embase <1974 to 2024 September 10>**

1 frail elderly/ or frailty/ 43608

2 frail*.ti,ab,kf. 63825

3 1 or 2 71263

4 "home monitoring".ti,ab,kf. 4018

5 health care management.ti,ab,kf. 1805

6 "care decision*".ti,ab,kf. 6873

7 risk assessment/ 774227

8 "health* monitoring".ti,ab,kf. 9240

9 patient monitoring.ti,ab,kf. 7684

10 telemonitoring/ 6543

11 telemedicine/ or telecare/ 51272

12 "patient specific model*ing".ti,ab,kf. 635

13 "digital* monitor*".ti,ab,kf. 479

14 remote sensing/ 17541

15 digital*.ti,ab,kf. 283967

16 digital twin/ 948

17 4 or 5 or 6 or 7 or 8 or 9 or 10 or 11 or 12 or 13 or 14 or 15 or 16 1143778

18 (Hospital adj2 at home).ti,ab,kf. 2359

19 "virtual ward".ti,ab,kf. 258

20 home.ti,ab,kf. 430755

21 community dwelling person/ 12574

22 independent living/ 8390

23 "acute care at home".ti,ab,kf. 58

24 "hospital in the home".ti,ab,kf. 380

25 "home hospital*".ti,ab,kf. 1061

26 "hospital-based home care".ti,ab,kf. 187

27 "rapid response".ti,ab,kf. 12354

28 home care/ 73255

29 domiciliary.ti,ab,kf. 3975

30 18 or 19 or 20 or 21 or 22 or 23 or 24 or 25 or 26 or 27 or 28 or 29 484082

31 3 and 17 and 30 1091

32 limit 31 to (english language and yr="2019 -Current") **678**

**Ovid MEDLINE(R) ALL <1946 to September 10, 2024>**

1 Frail Elderly/ or Frailty/ 23084

2 frail*.ti,ab,kf. 41509

3 1 or 2 46143

4 "home monitoring".ti,ab,kf. 2525

5 health care management.ti,ab,kf. 1498

6 "care decision*".ti,ab,kf. 5312

7 Risk Assessment/ 316976

8 "health* monitoring".ti,ab,kf. 9082

9 patient monitoring.ti,ab,kf. 5307

10 telecare.mp. 1035

11 Telemedicine/ or telemonitoring.mp. 42878

12 "patient specific model*ing".ti,ab,kf. 526

13 "digital* monitor*".ti,ab,kf. 327

14 Remote Sensing Technology/ 4411

15 digital*.ti,ab,kf. 229480

16 Digital twin.mp. 1055

17 4 or 5 or 6 or 7 or 8 or 9 or 10 or 11 or 12 or 13 or 14 or 15 or 16 608037

18 (Hospital adj2 at home).ti,ab,kf. 1567

19 "virtual ward".ti,ab,kf. 108

20 home.ti,ab,kf. 300300

21 Independent Living/ or community dwelling person.mp. 12883

22 "acute care at home".ti,ab,kf. 20

23 "hospital in the home".ti,ab,kf. 230

24 "home hospital*".ti,ab,kf. 672

25 "hospital-based home care".ti,ab,kf. 163

26 "rapid response".ti,ab,kf. 9632

27 Home Care Services/ 37290

28 domiciliary.ti,ab,kf. 2960

29 18 or 19 or 20 or 21 or 22 or 23 or 24 or 25 or 26 or 27 or 28 332401

30 3 and 17 and 29 521

31 limit 30 to (english language and yr="2019 -Current") **231**

**CINAHL (EBSCOhost)**

**Print Search History**

| Wed, September 11, 2024 03:47:53 pm     \| # \| Query \| Limiters/Expanders \| Results \| \| --- \| --- \| --- \| --- \| \| S29 \| S1 AND S27 AND S28 \| Limiters - Publication Date: 20190101-20241231 Expanders - Apply equivalent subjects Narrow by Language: - english Search modes - Proximity \| 439 \| \| S28 \| S14 OR S15 OR S16 OR S17 OR S18 OR S19 OR S20 OR S21 OR S22 OR S23 OR S24 OR S25 OR S26 \| Expanders - Apply equivalent subjects Search modes - Proximity \| 174,808 \| \| S27 \| S2 OR S3 OR S4 OR S5 OR S6 OR S7 OR S8 OR S9 OR S10 OR S11 OR S12 OR S13 \| Expanders - Apply equivalent subjects Search modes - Proximity \| 278,652 \| \| S26 \| AB domiciliary \| Expanders - Apply equivalent subjects Search modes - Proximity \| 662 \| \| S25 \| (MH "Home Health Care") OR "home care services" \| Expanders - Apply equivalent subjects Search modes - Proximity \| 26,848 \| \| S24 \| AB home care services \| Expanders - Apply equivalent subjects Search modes - Proximity \| 3,715 \| \| S23 \| AB "rapid response" \| Expanders - Apply equivalent subjects Search modes - Proximity \| 1,785 \| \| S22 \| AB "rapid response" \| Expanders - Apply equivalent subjects Search modes - Proximity \| 1,785 \| \| S21 \| AB "hospital-based home care" \| Expanders - Apply equivalent subjects Search modes - Proximity \| 63 \| \| S20 \| AB "home hospital*" \| Expanders - Apply equivalent subjects Search modes - Proximity \| 225 \| \| S19 \| AB "acute care at home" \| Expanders - Apply equivalent subjects Search modes - Proximity \| 55 \| \| S18 \| AB "hospital in the home" \| Expanders - Apply equivalent subjects Search modes - Proximity \| 101 \| \| S17 \| "community dwelling" OR (MH "Home Environment") \| Expanders - Apply equivalent subjects Search modes - Proximity \| 33,639 \| \| S16 \| AB home \| Expanders - Apply equivalent subjects Search modes - Proximity \| 132,332 \| \| S15 \| AB virtual ward \| Expanders - Apply equivalent subjects Search modes - Proximity \| 79 \| \| S14 \| AB hospital n2 at home \| Expanders - Apply equivalent subjects Search modes - Proximity \| 8,686 \| \| S13 \| TX digital twin* \| Expanders - Apply equivalent subjects Search modes - Proximity \| 135 \| \| S12 \| AB digital* \| Expanders - Apply equivalent subjects Search modes - Proximity \| 41,698 \| \| S11 \| AB remote sensing \| Expanders - Apply equivalent subjects Search modes - Proximity \| 199 \| \| S10 \| AB digital* monitor* \| Expanders - Apply equivalent subjects Search modes - Proximity \| 540 \| \| S9 \| AB patient specific model*ing \| Expanders - Apply equivalent subjects Search modes - Proximity \| 88 \| \| S8 \| SU ( telecare or telemedicine or telehealth ) OR SU telemonitoring \| Expanders - Apply equivalent subjects Search modes - Proximity \| 32,521 \| \| S7 \| AB patient monitoring \| Expanders - Apply equivalent subjects Search modes - Proximity \| 15,135 \| \| S6 \| AB "health* monitoring" \| Expanders - Apply equivalent subjects Search modes - Proximity \| 769 \| \| S5 \| (MH "Risk Assessment") \| Expanders - Apply equivalent subjects Search modes - Proximity \| 171,751 \| \| S4 \| AB care decision* \| Expanders - Apply equivalent subjects Search modes - Proximity \| 13,828 \| \| S3 \| AB health care management \| Expanders - Apply equivalent subjects Search modes - Proximity \| 7,786 \| \| S2 \| AB home monitoring \| Expanders - Apply equivalent subjects Search modes - Proximity \| 2,004 \| \| S1 \| (MM "Frail Elderly") OR ( frailty or frail* ) \| Expanders - Apply equivalent subjects Search modes - Proximity \| 23,558 \|         **Cochrane CENTRAL**    Search Name: SCR HaH DT  Date Run: 12/09/2024 11:21:58  Comment:    ID Search Hits  #1 MeSH descriptor: [Frailty] explode all trees 814  #2 MeSH descriptor: [Frail Elderly] explode all trees 1149  #3 frail* 6684  #4 #1 OR #2 OR #3 6684  #5 ("home monitoring"):ti,ab,kw OR ("health care management"):ti,ab,kw OR (care decision*):ti,ab,kw OR (health* monitoring):ti,ab,kw OR ("patient monitoring"):ti,ab,kw (Word variations have been searched) 63782  #6 MeSH descriptor: [Risk Assessment] explode all trees 13686  #7 MeSH descriptor: [Telemedicine] explode all trees 4989  #8 (telemonitoring):ti,ab,kw (Word variations have been searched) 1510  #9 (telecare):ti,ab,kw (Word variations have been searched) 287  #10 MeSH descriptor: [Remote Sensing Technology] explode all trees 75  #11 (digital*):ti,ab,kw (Word variations have been searched) 23148  #12 (digital twin):ti,ab,kw (Word variations have been searched) 88  #13 #5 OR #6 OR #7 OR #8 OR #9 OR #10 OR #11 OR #12 101470  #14 (hospital NEXT at home):ti,ab,kw (Word variations have been searched) 724  #15 ("virtual ward"):ti,ab,kw (Word variations have been searched) 11  #16 (home):ti,ab,kw (Word variations have been searched) 65276  #17 MeSH descriptor: [Independent Living] explode all trees 1177  #18 ("acute care at home"):ti,ab,kw (Word variations have been searched) 10  #19 ("hospital in the home"):ti,ab,kw (Word variations have been searched) 50  #20 (home NEXT hospital*):ti,ab,kw (Word variations have been searched) 173  #21 ("hospital-based home care"):ti,ab,kw (Word variations have been searched) 22  #22 ("rapid response"):ti,ab,kw (Word variations have been searched) 515  #23 MeSH descriptor: [Home Care Services] explode all trees 3132  #24 ("domiciliary"):ti,ab,kw (Word variations have been searched) 564  #25 #14 OR #15 OR #16 OR #17 OR #18 OR #19 OR #20 OR #21 OR #22 OR #23 OR #24 66968  #26 #4 AND #13 AND #25 with Cochrane Library publication date Between Jan 2019 and Sep 2024 **207** |
| --- | --- | --- | --- | --- | --- | --- | --- | --- | --- | --- | --- | --- | --- | --- | --- | --- | --- | --- | --- | --- | --- | --- | --- | --- | --- | --- | --- | --- | --- | --- | --- | --- | --- | --- | --- | --- | --- | --- | --- | --- | --- | --- | --- | --- | --- | --- | --- | --- | --- | --- | --- | --- | --- | --- | --- | --- | --- | --- | --- | --- | --- | --- | --- | --- | --- | --- | --- | --- | --- | --- | --- | --- | --- | --- | --- | --- | --- | --- | --- | --- | --- | --- | --- | --- | --- | --- | --- | --- | --- | --- | --- | --- | --- | --- | --- | --- | --- | --- | --- | --- | --- | --- | --- | --- | --- | --- | --- | --- | --- | --- | --- | --- | --- | --- | --- | --- | --- | --- | --- | --- |

Cochrane reviews = 4

Trials = **203**

**Web of Science**

(ALL=(frailty OR frail elderly)) OR TS=(frail*)

AND

TS=((“Home monitoring” OR “health care management” OR “care decisions” OR “health* monitoring” OR “patient monitoring” OR “telemonitoring” OR “telecare” OR “telemedicine” OR “Patient specific model*ing" OR “digital monitoring” OR “remote sensing” OR “digital*” OR “digital twin”))

AND

TS=((hospital NEAR/2 at home OR “virtual ward” OR Home OR “community dwelling person” OR independent living OR “acute care at home” OR “hospital in the home” OR “home hospital*” OR “hospital-based home care” OR “rapid response” OR home care OR domiciliary))

Results after limits 2019-2024 and English = **164**

**Scopus**

( ( TITLE-ABS-KEY ( frail* ) OR ALL ( frailty ) OR ALL ( frail AND elderly ) ) ) AND ( TITLE-ABS-KEY ( "Home monitoring" OR "health care management" OR "care decisions" OR "health* monitoring" OR "patient monitoring" OR "telemonitoring" OR "telecare" OR "telemedicine" OR "Patient specific model*ing" OR "digital monitoring" OR "remote sensing" OR "digital*" OR "digital twin" ) ) AND ( TITLE-ABS-KEY ( hospital W/2 at AND home OR "virtual ward" OR home OR "community dwelling person" OR independent AND living OR "acute care at home" OR "hospital in the home" OR "home hospital*" OR "hospital-based home care" OR "rapid response" OR home AND care OR domiciliary ) ) AND PUBYEAR > 2018 AND PUBYEAR < 2025 AND ( LIMIT-TO ( LANGUAGE , "English" ) )

Results = **25**

**Searches Updates 16.9.2025**

**Ovid EMBASE**

**Database:**
Embase <1974 to 2025 September 15>

| **#** | **Query** | **Results from 16 Sep 2025** |
| --- | --- | --- |
| 1 | frail elderly/ or frailty/ | 52,878 |
| 2 | frail*.ti,ab,kf. | 74,769 |
| 3 | 1 or 2 | 82,947 |
| 4 | "home monitoring".ti,ab,kf. | 4,758 |
| 5 | health care management.ti,ab,kf. | 1,909 |
| 6 | "care decision*".ti,ab,kf. | 7,486 |
| 7 | risk assessment/ | 807,067 |
| 8 | "health* monitoring".ti,ab,kf. | 11,507 |
| 9 | patient monitoring.ti,ab,kf. | 9,379 |
| 10 | telemonitoring/ | 8,220 |
| 11 | telemedicine/ or telecare/ | 58,476 |
| 12 | "patient specific model*ing".ti,ab,kf. | 693 |
| 13 | "digital* monitor*".ti,ab,kf. | 662 |
| 14 | remote sensing/ | 21,734 |
| 15 | digital*.ti,ab,kf. | 325,660 |
| 16 | digital twin/ | 1,744 |
| 17 | 4 or 5 or 6 or 7 or 8 or 9 or 10 or 11 or 12 or 13 or 14 or 15 or 16 | 1,231,489 |
| 18 | (Hospital adj2 at home).ti,ab,kf. | 2,720 |
| 19 | "virtual ward".ti,ab,kf. | 338 |
| 20 | home.ti,ab,kf. | 480,410 |
| 21 | community dwelling person/ | 14,100 |
| 22 | independent living/ | 9,568 |
| 23 | "acute care at home".ti,ab,kf. | 65 |
| 24 | "hospital in the home".ti,ab,kf. | 420 |
| 25 | "home hospital*".ti,ab,kf. | 1,228 |
| 26 | "hospital-based home care".ti,ab,kf. | 191 |
| 27 | "rapid response".ti,ab,kf. | 13,987 |
| 28 | home care/ | 77,128 |
| 29 | domiciliary.ti,ab,kf. | 4,185 |
| 30 | 18 or 19 or 20 or 21 or 22 or 23 or 24 or 25 or 26 or 27 or 28 or 29 | 538,295 |
| 31 | 3 and 17 and 30 | 1,319 |
| 32 | limit 31 to (english language and yr="2019 -Current") | 877 |
| 33 | limit 32 to dc=20240911-20250916 | 218 |

**Database:**
**Ovid MEDLINE(R)** ALL <1946 to September 15, 2025>

| **#** | **Query** | **Results from 16 Sep 2025** |
| --- | --- | --- |
| 1 | Frail Elderly/ or Frailty/ | 25,472 |
| 2 | frail*.ti,ab,kf. | 47,557 |
| 3 | 1 or 2 | 52,190 |
| 4 | "home monitoring".ti,ab,kf. | 2,724 |
| 5 | health care management.ti,ab,kf. | 1,571 |
| 6 | "care decision*".ti,ab,kf. | 5,695 |
| 7 | Risk Assessment/ | 333,277 |
| 8 | "health* monitoring".ti,ab,kf. | 11,163 |
| 9 | patient monitoring.ti,ab,kf. | 6,160 |
| 10 | telecare.mp. | 1,092 |
| 11 | Telemedicine/ or telemonitoring.mp. | 47,745 |
| 12 | "patient specific model*ing".ti,ab,kf. | 583 |
| 13 | "digital* monitor*".ti,ab,kf. | 417 |
| 14 | Remote Sensing Technology/ | 4,927 |
| 15 | digital*.ti,ab,kf. | 258,237 |
| 16 | Digital twin.mp. | 1,629 |
| 17 | 4 or 5 or 6 or 7 or 8 or 9 or 10 or 11 or 12 or 13 or 14 or 15 or 16 | 659,633 |
| 18 | (Hospital adj2 at home).ti,ab,kf. | 1,739 |
| 19 | "virtual ward".ti,ab,kf. | 125 |
| 20 | home.ti,ab,kf. | 318,603 |
| 21 | Independent Living/ or community dwelling person.mp. | 14,522 |
| 22 | "acute care at home".ti,ab,kf. | 27 |
| 23 | "hospital in the home".ti,ab,kf. | 247 |
| 24 | "home hospital*".ti,ab,kf. | 730 |
| 25 | "hospital-based home care".ti,ab,kf. | 165 |
| 26 | "rapid response".ti,ab,kf. | 10,935 |
| 27 | Home Care Services/ | 38,459 |
| 28 | domiciliary.ti,ab,kf. | 3,021 |
| 29 | 18 or 19 or 20 or 21 or 22 or 23 or 24 or 25 or 26 or 27 or 28 | 353,423 |
| 30 | 3 and 17 and 29 | 616 |
| 31 | limit 30 to (english language and yr="2019 -Current") | 326 |
| 32 | limit 31 to dt=20240911-20250916 | 84 |

**EBSCOhost CINAHL**

Date Run: 17/09/2025

all (AB (digital* OR “remote sensing” OR ”digital* monitor*” OR “patient specific model*ing” OR patient monitoring OR "health* monitoring" OR care decision* OR “health care management” OR “home monitoring”) OR SU (telecare OR telemedicine or telehealth OR telemonitoring) OR (MH "Risk Assessment") OR (TX digital twin*)) AND all (AB (domiciliary OR “home care services” OR "rapid response" OR "hospital-based home care" OR "home hospital*" OR "acute care at home" OR "hospital in the home" OR "community dwelling" OR home OR “virtual ward” OR "home care services" OR (hospital n2 at home)) OR (MH "Home Health Care" OR "Home Environment")) AND all ((MM "Frail Elderly") OR ( frailty or frail* ))

Limits 11/09/2024-16/09/25

Limits English

**Results 27**

**COCHRANE**

Search Name: SCR HaH DT Updated Sept 2025

Date Run: 17/09/2025 17:30:14

Comment:

ID Search Hits

#1 MeSH descriptor: [Frailty] explode all trees 962

#2 MeSH descriptor: [Frail Elderly] explode all trees 1216

#3 frail* 7365

#4 #1 OR #2 OR #3 7365

#5 ("home monitoring"):ti,ab,kw OR ("health care management"):ti,ab,kw OR (care decision*):ti,ab,kw OR (health* monitoring):ti,ab,kw OR ("patient monitoring"):ti,ab,kw (Word variations have been searched) 68175

#6 MeSH descriptor: [Risk Assessment] explode all trees 13634

#7 MeSH descriptor: [Telemedicine] explode all trees 5614

#8 (telemonitoring):ti,ab,kw (Word variations have been searched) 1591

#9 (telecare):ti,ab,kw (Word variations have been searched) 317

#10 MeSH descriptor: [Remote Sensing Technology] explode all trees 78

#11 (digital*):ti,ab,kw (Word variations have been searched) 26609

#12 (digital twin):ti,ab,kw (Word variations have been searched) 115

#13 #5 OR #6 OR #7 OR #8 OR #9 OR #10 OR #11 OR #12 109167

#14 (hospital NEXT at home):ti,ab,kw (Word variations have been searched) 766

#15 ("virtual ward"):ti,ab,kw (Word variations have been searched) 13

#16 (home):ti,ab,kw (Word variations have been searched) 68858

#17 MeSH descriptor: [Independent Living] explode all trees 1323

#18 ("acute care at home"):ti,ab,kw (Word variations have been searched) 10

#19 ("hospital in the home"):ti,ab,kw (Word variations have been searched) 50

#20 (home NEXT hospital*):ti,ab,kw (Word variations have been searched) 175

#21 ("hospital-based home care"):ti,ab,kw (Word variations have been searched) 21

#22 ("rapid response"):ti,ab,kw (Word variations have been searched) 542

#23 MeSH descriptor: [Home Care Services] explode all trees 3164

#24 ("domiciliary"):ti,ab,kw (Word variations have been searched) 563

#25 #14 OR #15 OR #16 OR #17 OR #18 OR #19 OR #20 OR #21 OR #22 OR #23 OR #24 70683

#26 #4 AND #13 AND #25 with Cochrane Library publication date Between Sep 2024 and Sep 2025 **43**

**Web of Science**

ALL= (frailty OR frail elderly) OR (frail*)

AND

(“Home monitoring” OR “health care management” OR “care decisions” OR “health* monitoring” OR “patient monitoring” OR “telemonitoring” OR “telecare” OR “telemedicine” OR “Patient specific model*ing" OR “digital monitoring” OR “remote sensing” OR “digital*” OR “digital twin”)

AND

(hospital NEAR/2 at home OR “virtual ward” OR Home OR “community dwelling person” OR independent living OR “acute care at home” OR “hospital in the home” OR “home hospital*” OR “hospital-based home care” OR “rapid response” OR home care OR domiciliary)

Date= 2024-09-11 to 2025-09-16

Limit = English

Results = 46

**Scopus**

( TITLE-ABS-KEY ( frailty OR "frail elderly" OR frail* ) AND TITLE-ABS-KEY ( "Home monitoring" OR "health care management" OR "care decisions" OR "health* monitoring" OR "patient monitoring" OR telemonitoring OR telecare OR telemedicine OR "Patient specific model*ing" OR "digital monitoring" OR "remote sensing" OR digital* OR "digital twin" ) AND TITLE-ABS-KEY ( ( ( hospital W/2 at AND home ) OR "virtual ward" OR home OR "community dwelling person" OR ( independent AND living ) OR "acute care at home" OR "hospital in the home" OR "home hospital*" OR "hospital-based home care" OR "rapid response" OR ( home AND care ) OR domiciliary ) ) ) AND ORIG-LOAD-DATE > 20240911 AND PUBYEAR > 2023 AND PUBYEAR < 2026 AND ( LIMIT-TO ( LANGUAGE , "English" ) )

Results = 86
