# Supplementary material for: Development of a Hospital-at-Home Digital Twin for Patients With Frailty: Scoping Review
Source: J Med Internet Res. 2025 Dec 10;27:e81510. doi: 10.2196/81510 (PMC12694950; doi:10.2196/81510)
Supplement: Multimedia Appendix 3 [file jmir-v27-e81510-s003.docx]

| Table 1. Study Characteristics (separated by evidence type) **Studies using Quantitative methods** | | | | |  |  |
| --- | --- | --- | --- | --- | --- | --- |
| **Author, Year** | **Country** | **Type of paper** | **Evidence type** | **Research design** | **Population** | **Concept** |
| Bharadwaj, 2023 [37] | USA | Journal article | Quantitative | Retrospective observational cohort study | Older adults with frailty | Advanced Health Care Practitioner-Led Home-Visit Primary Care Program |
| Kobulnik, 2022 [38] | Canada | Journal article | Quantitative | Quasi-experimental cohort study | Homebound, frail older adults | Assisted virtual care model |
| Bian, 2022 [67] | Canada | Journal article | Quantitative | Experimental validation study | Older adults (pre-frail) | Sensor-based system (Frailty toolkit) |
| Spangler, 2024 [39] | USA | Journal article | Quantitative | Observational descriptive validation study | Frail and pre-frail community-dwelling adults recently discharged from hospital | multimodal movement monitoring system |
| Lette, 2022 [40] | The Netherlands | Journal article | Quantitative | Observational Cross-sectional study | Frail older people | InterRAI-HC assessment using a standardised and fully structured CGA instrument. |
| Perman, 2021 [41] | Argentina | Journal article | Quantitative | Quasi-experimental | Home dwelling frail older persons | Health and social care integration programme |
| Jepma, 2021b [43] | The Netherlands | Journal article | Quantitative | RCT | As per study above | As per study above |
| Pérez Bazán, 2019 [46] | Spain | Journal article | Quantitative | Interventional cohort study | Frail older adults | Multi-disciplinary intervention integrating primary care, geriatric care and community services. (+AGIL) |
| Schoon, 2020 [48] | The Netherlands | Journal article | Quantitative | RCT (pilot feasibility) | Older persons at risk of falling (16.7% confirmed frail) | self-management fall prevention program |
| Veyron, 2019 [49] | France | Journal article | Quantitative | Observational cohort study | Frail elderly | Home care aides (Home visits) reporting functional status on smartphone application |
| Ohta, 2024 [51] | Japan | Journal article | Quantitative | Feasibility study | Frail or pre-frail older adults | Mobile  Health Application "Online Kayoinoba" |
| Camerlingo, 2023 [53] | USA | Journal article | Quantitative | Cross-sectional Feasibility study | Healthy frail community-dwelling older adults | Digital health technologies |
| Wuestney, 2023 [54] | USA | Conference paper | Quantitative | Case study | frail older adult (83-year-old female resident) | Measuring the complexity of human indoor activity using smart home sensor data, as a potential digital biomarker for detecting change in a person's frailty. |
| Okpara, 2023 [94] | Canada | Journal article | Quantitative | Feasibility RCT | Older adults with frailty | Geras virtual frailty rehabilitation program |
| Nagatomi, 2022 [95] | Japan | Journal article | Quantitative | RCT | heart failure patients with frailty | home-based cardiac rehabilitation (HBCR) program |
| Pérez-Rodríguez, 2021 [55] | Spain | Journal article | Quantitative | RCT (pilot feasibility)  (Usability and user- experience evaluation) | Frail and pre-frail older adults | CAPACITY: A technological Ecosystem for remote follow-up of frailty |
| Pérez-Rodríguez, 2020 [56] | Spain | Journal article | Quantitative | RCT (pilot) | frail and pre-frail diabetic older population | Substudy of above- using CAPACITY ecosystem |
| Rens, 2021 [85] | USA | Journal article | Quantitative | Longitudinal observational study | Ambulatory patients with CAD who were scheduled for coronary artery bypass graft (CABG). (Mean age 68.9 and had comorbidities) | home-based 6MWT using app in iPhone and Apple Watch |
| Baek, 2022 [97] | Korea | Journal article | Quantitative | Feasibility study (usability) | Frail and pre-frail older adults | Integrated home IoT service |
| Sarkar, 2022 [60] | Canada | Journal article | Quantitative | Feasibility pilot study (Non-RCT) | Frail adults (over 55yrs) | Enhanced Telehealth home-monitoring intervention after cardiac surgery |
| De Luca, 2021 [61] | Italy | Journal article | Quantitative | RCT | Frail older adults | multi-disciplinary telemedicine service |
| Calvillo-arbizu, 2021 [72] | Spain | Journal article | Quantitative | Feasibility pilot study | Frail and pre-frail older adults | Sensor based mHealth platform integrated in a service-based architecture inside the FRAIL project. |
| Soufian, 2022 [87] | UK | Journal article | Quantitative | Experimental validation study | Older adults with frailty | MS Kinect Fall detection system |
| Fayad, 2019 [73] | France | Conference paper | Quantitative | Experimental pilot study | Frail elderly but piloted on young healthy volunteers | Fall detection application for the elderly |
| Infarinato, 2020 [62] | Austria, Denmark, Italy, and the Netherlands | Journal article | Quantitative | longitudinal study | Frail adults | eWALL platform in patients home |
| Razjouyan, 2020 [79] | USA | Journal article | Quantitative | Observational study | Older adults with physical frailty | Remote physical activity (PA) monitoring using a pendant sensor |
| De Cola, 2020 [63] | Italy | Journal article | Quantitative | Descriptive exploratory study (usability) | Frail elderly | Novel telemedicine system, teleassistance program, which included remote surveillance and tele-counselling services with different HCPs |
| Bruns, 2019 [105] | The Netherlands | Journal article | Quantitative | Pilot Feasibility study | Frail elderly planned for colorectal cancer surgery | Fit4SurgeryTV at-home prehabilitation |
| Chkeir, 2019 [80] | France | Journal article | Quantitative | Pilot study (correlation) | Frail elderly | ARPEGE Pack |
| Hamada, 2020 [81] | Japan | Conference paper | Quantitative | Case Study | Single elderly participant | Ambient health monitoring handrail sensor device on stairway |
| Maekawa and Kume, 2025 [89] | Japan | Journal article | Quantitative | Cross-sectional study | Community dwelling older adults with social pre-frailty and social frailty | Fitbit device |
| Li, 2025 [90] | China | Journal article | Quantitative | RCT | Community-dwelling older adults (prefrail and frail) | mobile health application and wearable devices for continuous monitoring |
| Huang, 2025 [91] | Taiwan | Journal article | Quantitative | Feasibility study | Community dwelling frail older adults | VR-based system incorporating skeletal visual feedback (Home-based exercise program) |
| Dodson, 2025 [100] | USA | Journal article | Quantitative | RCT | Aged 65 years or older and had an acute  event related to ischemic heart disease. Most patients had frailty or prefrailty. | mHealth-CR programn(software, counselling and physiological monitoring) |
| Nejadshamsi, 2025 [101] | Canada | Journal article | Quantitative | Feasibility study | Community-dwelling older adults (50% with frailty) | Wi-Fi–based motion sensors. HOPE (Home-Based Older Adults’ Depression Prediction) model |
| Valdés-Aragonés, 2024 [92] | Spain | Journal article | Quantitative | Pilot RCT | Older adults aged ≥70 years meeting 2 or more Fried frailty phenotype  criteria, having 4 or more comorbidities | FACET monitoring system |
| HomeLink Healthcare, 2023 [103] | UK | Report | Quantitative | Case study | Patients with frailty and patients with complex needs | Four virtual wards across an integrated care system |
| **Studies using Qualitative methods** | | |  |  |  |  |
| **Author, Year** | **Country** | **Type of paper** | **Evidence type** | **Research design** | **Population** | **Concept** |
| Sepehri, 2022 [77] | Canada | Journal article | Qualitative | Observational descriptive Case Report | Home-living older adults requiring frailty assessment | Electronic tool to assess and manage patients with frailty |
| Piau, 2021 [78] | France | Journal article | Qualitative | RCT | Frail older adults (frail and pre-frail) | wearable insole for monitoring |
| Terbraak, 2023 [71] | The Netherlands | Journal article | Qualitative | RCT | Frail older patients | Home -based Cardiac Rehabilitation (CR) |
| Jepma, 2021a [109] | The Netherlands | Journal article | Qualitative | RCT | Frail older cardiac patients | Transitional care intervention |
| Canet- Vélez, 2023 [45] | Spain | Journal article | Qualitative | Descriptive | Frail older adults living in the community | A multicomponent care intervention- "+AGIL" Barcelona programme |
| Chang, 2023 [102] | Taiwan | Journal article | Qualitative | Feasibility study (usability) | Frail older adults | Novel smart somatosensory wearable assistive device (SSWAD) during home rehabilitation |
| Timm, 2024 [52] | Sweden | Journal article | Qualitative | Exploratory study | Frail and pre-frail older adults | Part of larger research project - POSITIVE system (promote physical exercise in home environment) |
| Marinello, 2021 [57] | Italy | Journal article | Qualitative | Case study | 91yr old frail woman with severe dementia, COVID-19 and multiple comorbidities. | Telemedicine-assisted care in HaH setting |
| Glomsås, 2022 [96] | Norway | Journal article | Qualitative | Exploratory study (Descriptive) | Family caregivers of frail older adults | Home care services supported by welfare technology. |
| Weeks, 2022 [86] | Canada | Journal article | Qualitative | RCT | Frail older adults (family and friend caregivers) | Passive remote monitoring technology in home care services |
| Toufik, 2019 [82] | France | Conference paper | Qualitative | Longitudinal study | Pre-frail older adults | Home monitoring system (Fragil-IT system) using sensors |
| Jo, 2021[68] | Korea | Journal article | Qualitative | Exploratory study | Frail elderly | Internet of things-based Integrated smart home system (ISHS) |
| British Red Cross, 2024 [65] | UK | Report | Qualitative | Evaluation | Frailty, and all virtual wards | A number of virtual wards in the UK and VCSE organisations |
| **Studies using mixed methods** | | |  |  |  |  |
| **Author, Year** | **Country** | **Type of paper** | **Evidence type** | **Research design** | **Population** | **Concept** |
| King, 2020 [106] | Canada | Journal article | Mixed methods | Observational study | Frail seniors | Home bathing |
| Cobo, 2020 [83] | Spain | Journal article | Mixed methods | Observational study | Older adults at risk of frailty | Low-cost-senor to count sit-to-stand transitions combined with android app |
| Fristedt, 2019 [42] | Sweden | Journal article | Mixed methods | RCT | Community-dwelling frail older adults | Mobile Geriatric Team (MGT) |
| Nixon, 2021 [93] | UK | Journal article | Mixed methods | RCT (pilot) | Frail and pre-frail older adults with CKD | EX-FRAIL CKD Trial - home-based exercise intervention |
| Villalba-Mora, 2021 [66] | Spain | Journal article | Mixed methods | Exploratory study (user centred design) | Older adults aged >/70 years old, living in own home, with family support, and ability to walk with or without technical assistance. | home monitoring system (HMS) that supports CGA in a patient's home. |
| Olde Keizer, 2019 [44] | The Netherlands | Journal article | Mixed methods | Observational study | Frail or pre-frail older adults | Socially assistive robot |
| Rodrigues, 2023 [47] | Canada | Journal article | Mixed methods | Feasibility longitudinal study | Frail or pre-frail older adults | Mapping sedentary behaviour using wearable sensors |
| Vaz, 2022 [50] | Australia | Journal article | Mixed methods | Pre-post intervention study | Frail older adults | prescribing tailored home exercise program using a tailored self-modelled video |
| Belmin, 2022 [58] | France | Journal article | Mixed methods | Multi-centre uncontrolled pragmatic trial | Community-dwelling frail older adults reeiving support from home aides (HAs) | An eHealth system that produces an alert for a high risk of ED visits. |
| Diamond, 2021 [59] | USA | Journal article | Mixed methods | Pilot prospective multicentre cohort study | Frail post-transplant patients | Mobile health technology post-transplant |
| Joddrell, 2021 [88] | UK | Journal article | Mixed methods | Longitudinal Feasibility study | Frail and pre-frail older adults | Continuous in-home walking speed monitoring- ambient sensor |
| Fowler-Davis, 2020 [98] | UK | Journal article | Mixed methods | Pilot study | Frail older adults (people with dementia who live alone) and their family carer | Digital device 3Rings™ utility monitoring in the home |
| Zacharaki, 2020 [74] | Greece, France, Cyprus | Journal article | Mixed methods | Proof of concept Evaluation study | Frail and pre-frail older adults | FrailSafe System ICT platform for unobtrusive sensing of multi-domain frailty |
| Aylward-Wotton, 2025 [108] | UK | Journal article | Mixed methods | Case Study | Adults with long-term conditions, including frailty, and Rockwood frailty index with a high-risk classification defined as Level 5 and above. | Continuous pressure monitoring (CPM) |
| Elliot, 2021[64] | UK | Report | Mixed methods | Evaluation | Patients with severe or moderate frailty and with an urgent medical need | Virtual ward (VW) service |
| Player, 2023 [75] | UK | Report | Mixed methods | Evaluation | Older people with frailty | Digital remote monitoring enabled frailty VW |
| Edwards, 2024 [76] | UK | Report | Mixed methods | Evaluation | 31% frailty and various VWs | 29 virtual wards in the southeast region |
| Dawe, 2022 [69] | UK | Report | Mixed methods | Evaluation | Frailty and Respiratory | Virtual wards across an ICS |
| NHSE, 2022 [70] | UK | Report | Mixed methods | Evaluation | All patients supported by remote monitoring including patients with frailty | Remote monitoring technologies within London, including virtual wards |
