# Supplementary material for: Development of a Hospital-at-Home Digital Twin for Patients With Frailty: Scoping Review
Source: J Med Internet Res. 2025 Dec 10;27:e81510. doi: 10.2196/81510 (PMC12694950; doi:10.2196/81510)
Supplement: Multimedia Appendix 4 [file jmir-v27-e81510-s004.docx]

| **Applications of the tool(s)**  First author, date | Care Communication & Coordination | | | | Clinical monitoring & review | | | Monitoring frailty markers & physical function | | | | Improving patient outcomes | | | | | | Early deterioration alert | | Environmental monitoring | | | Education & training | |
| --- | --- | --- | --- | --- | --- | --- | --- | --- | --- | --- | --- | --- | --- | --- | --- | --- | --- | --- | --- | --- | --- | --- | --- | --- |
|  | Service coordination | Real-time data communication | Create connections among parties | Monitoring social interactions | Clinical Review | Vital Signs Monitoring | Medication Review | Monitoring behavioural change | Assessing change in frailty status | Measuring change in function/ADL | Monitoring nutrition | Improving ADL/ muscle strength/ physical function | Assisting with functional activities (bathing) | Improving health outcomes | Supporting improvement in mental health & cognitive function | Improving nutrition | Personalising intervention/ care plan | Identify/ Alerting to early deterioration | Falls detection | Monitoring patient location | Supporting patient welfare / safety | Monitoring the environment | Patient education | Caregiver training & support |
| Bharadwaj, 2023 [37] | X |  |  |  |  |  |  |  |  |  |  |  |  |  |  |  |  |  |  |  |  |  |  |  |
| Kobulnik, 2022 [38] |  |  |  |  | X | X | X |  |  |  |  |  |  |  |  |  |  |  |  |  |  |  |  |  |
| Bian, 2022 [67] |  | X |  |  |  | X |  | X | X | X |  |  |  |  |  |  |  |  |  |  |  | X |  |  |
| Sepehri, 2022 [77] |  |  |  |  |  |  |  | X | X |  |  |  |  |  |  |  |  |  |  |  |  |  |  |  |
| Spangler, 2024 [39] |  |  |  |  |  |  |  |  |  | X |  |  |  |  |  |  |  |  |  |  |  |  |  |  |
| Lette, 2022 [40] |  |  |  |  |  |  |  |  | X |  |  |  |  |  |  |  |  |  |  |  |  |  |  |  |
| King, 2020 [106] |  |  |  |  |  |  |  |  |  |  |  |  | X |  |  |  |  |  |  |  |  |  |  |  |
| Perman, 2021 [41] |  |  |  |  |  |  |  |  |  | X |  |  |  |  |  |  |  |  |  |  |  |  |  |  |
| Piau, 2021 [78] |  |  |  |  |  |  |  |  |  | X |  |  |  |  |  |  |  |  |  |  |  |  |  |  |
| Cobo, 2020 [83] |  |  |  |  |  |  |  |  | X | X |  |  |  |  |  |  | X |  |  |  |  |  |  |  |
| Fristedt, 2019 [42] |  |  |  |  |  |  |  |  | X |  |  |  |  |  |  |  |  |  |  |  |  |  |  |  |
| Terbraak, 2023 [71] |  |  |  |  |  | X |  |  |  |  |  | X |  |  |  |  |  |  |  |  |  |  |  |  |
| Jepma, 2021a [109] |  |  |  |  |  |  |  |  |  |  |  |  |  |  |  |  | X | X |  |  |  |  |  |  |
| Jepma, 2021b [43] |  |  |  |  |  |  |  |  |  |  |  |  |  |  |  |  | X | X |  |  |  |  |  |  |
| Nixon, 2021 [93] |  |  |  |  |  |  |  |  |  |  |  | X |  | X |  |  |  |  |  |  |  |  |  |  |
| Villalba-Mora, 2021 [66] |  |  |  |  |  |  |  |  | X |  |  |  |  |  |  |  |  |  |  |  |  |  |  |  |
| Olde Keizer, 2019 [44] |  |  |  |  |  |  |  |  | X |  |  | X |  |  |  |  |  |  |  |  |  |  |  |  |
| Canet- Vélez, 2023 [45] |  |  |  |  | X |  | X |  |  |  |  | X |  |  |  |  |  |  |  |  |  |  |  |  |
| Pérez Bazán, 2019 [46] |  |  |  |  |  |  |  |  |  |  |  | X |  |  |  | X |  |  |  |  |  |  | X |  |
| Rodrigues, 2023 [47] |  |  |  |  |  |  |  | X |  | X |  |  |  |  |  |  |  |  |  | X |  |  |  |  |
| Schoon, 2020 [48] |  |  |  |  |  |  |  |  |  | X |  |  |  |  |  |  |  |  |  |  |  |  |  |  |
| Veyron, 2019 [49] |  |  |  |  |  |  |  |  |  | X |  |  |  |  |  |  |  |  |  |  |  |  |  |  |
| Vaz, 2022 [50] |  |  |  |  |  |  |  |  |  |  |  | X |  | X |  |  |  |  |  |  |  |  |  |  |
| Chang, 2023 [102] |  |  |  |  |  |  |  |  |  |  |  | X |  |  |  |  |  |  |  |  |  |  |  |  |
| Ohta, 2024 [51] |  |  |  |  |  |  |  |  |  |  |  | X |  | X |  |  |  |  |  |  |  |  |  |  |
| Timm, 2024 [52] |  |  |  |  |  |  |  |  |  | X |  | X |  |  |  |  |  |  |  |  |  |  |  |  |
| Camerlingo, 2023 [53] |  |  |  |  |  | X |  |  |  | X | X |  |  |  |  | X |  |  |  |  |  |  |  |  |
| Wuestney, 2023 [54] |  |  |  |  |  |  |  |  | X | X |  |  |  |  |  |  |  |  |  |  |  |  |  |  |
| Okpara, 2023 [94] |  |  |  |  |  |  |  |  |  |  |  | X |  | X |  |  |  |  |  |  |  |  |  |  |
| Nagatomi, 2022 [95] |  |  |  |  |  |  |  |  |  |  |  | X |  | X |  |  |  |  |  |  |  |  | X |  |
| Pérez-Rodríguez, 2021 [55] |  | X | X |  |  |  |  |  |  |  |  |  |  |  |  |  | X | X |  |  |  |  |  |  |
| Pérez-Rodríguez, 2020 [56] |  |  |  |  |  |  |  |  |  | X |  |  |  |  |  |  | X | X |  |  |  |  |  |  |
| Marinello, 2021 [57] |  | X | X |  | X |  |  |  |  |  |  |  |  |  |  |  |  |  |  |  |  |  |  | X |
| Belmin, 2022 [58] |  |  |  |  |  |  |  |  |  |  |  |  |  |  |  |  |  | X |  |  |  |  |  |  |
| Diamond, 2021 [59] |  |  |  |  |  |  |  |  |  |  |  | X |  |  |  |  |  |  |  |  |  |  |  |  |
| Rens, 2021 [85] |  |  |  |  |  |  |  |  |  | X |  |  |  |  |  |  |  |  |  |  |  |  |  |  |
| Glomsås , 2022 [96] |  |  |  |  |  |  |  |  |  |  |  | X |  |  |  |  |  |  |  |  | X |  |  |  |
| Baek, 2022 [97] |  |  |  |  |  |  |  |  |  |  |  | X |  |  |  |  |  | X |  |  | X |  |  |  |
| Sarkar, 2022 [60] |  |  |  |  |  | X |  |  |  |  |  |  |  |  |  |  |  |  |  |  |  |  |  |  |
| De Luca, 2021 [61] |  |  |  |  | X |  |  |  |  | X |  |  |  |  | X |  |  |  |  |  |  |  |  |  |
| Calvillo-arbizu, 2021 [72] |  |  |  |  |  | X |  |  |  | X |  |  |  |  |  |  | X |  | X |  |  |  |  |  |
| Weeks, 2022 [86] |  |  |  |  |  | X |  | X |  | X |  |  |  |  |  |  |  |  | X |  |  |  |  |  |
| Soufian, 2022 [87] |  |  |  |  |  |  |  |  |  | X |  |  |  |  |  |  |  |  |  |  |  |  |  |  |
| Joddrell, 2021 [88] |  |  |  |  |  |  |  |  |  | X |  |  |  |  |  |  |  |  |  |  |  |  |  |  |
| Fayad, 2019 [73] |  |  |  |  |  | X |  |  |  |  |  |  |  |  |  |  |  |  | X |  |  |  |  |  |
| Infarinato, 2020 [62] |  |  |  |  |  | X |  | X |  |  |  |  |  |  |  |  |  |  |  |  |  | X |  |  |
| Razjouyan, 2020 [79] |  |  |  |  |  |  |  |  | X | X |  |  |  |  |  |  |  |  |  |  |  |  |  |  |
| De Cola, 2020 [63] |  | X |  |  | X | X |  |  |  |  |  |  |  |  |  |  |  |  |  |  |  |  |  |  |
| Bruns, 2019 [105] |  |  |  |  |  |  |  |  |  |  |  | X |  |  |  | X |  |  |  |  |  |  |  |  |
| Chkeir, 2019 [80] |  |  |  |  |  |  |  |  | X | X |  |  |  |  |  |  |  |  |  |  |  |  |  |  |
| Hamada, 2020 [81] |  |  |  |  |  |  |  |  | X | X |  |  |  |  |  |  |  |  |  |  |  |  |  |  |
| Fowler-Davis, 2020 [98] |  |  |  |  |  |  |  |  |  | X |  |  |  |  |  |  |  |  |  |  |  |  |  |  |
| Zacharaki, 2020 [74] |  |  |  | X |  | X |  | X | X | X |  | X |  |  | X |  |  |  |  |  |  |  |  |  |
| Toufik, 2019 [82] |  |  |  |  |  |  |  | X | X | X |  |  |  |  |  |  |  |  |  |  |  | X |  |  |
| Jo, 2021[68] |  | X |  |  |  | X |  | X |  | X |  |  |  |  |  |  |  |  | X | X |  | X |  |  |
| Maekawa and Kume, 2025 [89] |  |  |  |  | X |  |  |  |  | X |  |  |  |  |  |  |  |  |  |  |  |  |  |  |
| Aylward-Wotton, 2025 [108] |  |  |  |  |  |  |  |  |  |  |  |  |  |  |  |  |  | X |  |  |  |  |  |  |
| Li, 2025 [90] |  |  |  |  |  |  |  |  |  | X |  |  |  |  |  |  | X |  |  |  |  |  |  |  |
| Huang, 2025 [91] |  |  |  |  |  |  |  |  |  |  |  | X |  |  |  |  |  |  |  |  |  |  | X |  |
| Dodson, 2025 [100] |  |  |  |  |  |  |  |  |  | X |  | X |  |  |  |  |  |  |  |  |  |  |  |  |
| Nejadshamsi, 2025 [101] |  |  |  |  |  |  |  | X |  | X |  |  |  |  |  |  |  |  |  |  |  |  |  |  |
| Valdés-Aragonés, 2024 [92] |  |  | X |  |  |  |  |  | X |  | X |  |  |  |  |  |  | X |  |  |  |  |  |  |
| Elliot, 2021[64] | X |  |  |  | X | X |  |  |  |  |  |  |  |  |  |  |  |  |  |  |  |  |  |  |
| Player, 2023 [75] |  |  |  |  |  | X |  |  |  |  |  |  |  |  |  |  |  |  |  |  |  | X |  |  |
| British Red Cross, 2024 [65] |  |  |  |  | X |  |  |  |  |  |  |  |  |  |  |  |  |  |  |  | X | X |  |  |
| Edwards, 2024 [76] |  |  |  |  | X | X |  |  |  |  |  |  |  |  |  |  |  | X |  |  |  |  |  |  |
| Dawe, 2022 [69] | X | X |  |  | X | X |  |  |  |  |  |  |  |  |  |  |  |  |  |  |  |  |  |  |
| HomeLink Healthcare, 2023 [103] |  |  |  |  |  |  |  |  |  |  |  |  |  |  |  |  |  |  |  |  |  |  |  |  |
| NHSE, 2022 [70] | X | X |  |  | X | X |  |  |  |  |  |  |  |  |  |  |  | X |  |  |  |  | X |  |

Abbreviations:

ADL = Activities of daily living
